# Integrative single-cell omics analyses reveal epigenetic heterogeneity in mouse embryonic stem cells

**DOI:** 10.1371/journal.pcbi.1006034

**Published:** 2018-03-21

**Authors:** Yanting Luo, Jianlin He, Xiguang Xu, Ming-an Sun, Xiaowei Wu, Xuemei Lu, Hehuang Xie

**Affiliations:** 1 Key Laboratory of Genomic and Precision Medicine, Beijing Institute of Genomics, Chinese Academy of Sciences, Beijing, China; 2 CAS Center for Excellence in Animal Evolution and Genetics, Chinese Academy of Sciences, Kunming, China; 3 University of Chinese Academy of Sciences, Beijing, China; 4 Epigenomics and Computational Biology Lab, Biocomplexity Institute of Virginia Tech, Blacksburg, United States of America; 5 Department of Biological Sciences, Virginia Tech, Blacksburg, United States of America; 6 Department of Statistics, Virginia Tech, Blacksburg, United States of America; 7 Department of Biomedical Sciences and Pathobiology, Virginia-Maryland College of Veterinary Medicine, Virginia Tech, Blacksburg, United States of America; Ottawa University, CANADA

## Abstract

Embryonic stem cells (ESCs) consist of a population of self-renewing cells displaying extensive phenotypic and functional heterogeneity. Research towards the understanding of the epigenetic mechanisms underlying the heterogeneity among ESCs is still in its initial stage. Key issues, such as how to identify cell-subset specifically methylated loci and how to interpret the biological meanings of methylation variations remain largely unexplored. To fill in the research gap, we implemented a computational pipeline to analyze single-cell methylome and to perform an integrative analysis with single-cell transcriptome data. According to the origins of variation in DNA methylation, we determined the genomic loci associated with allelic-specific methylation or asymmetric DNA methylation, and explored a beta mixture model to infer the genomic loci exhibiting cell-subset specific methylation (CSM). We observed that the putative CSM loci in ESCs are significantly enriched in CpG island (CGI) shelves and regions with histone marks for promoter and enhancer, and the genes hosting putative CSM loci show wide-ranging expression among ESCs. More interestingly, the putative CSM loci may be clustered into co-methylated modules enriching the binding motifs of distinct sets of transcription factors. Taken together, our study provided a novel tool to explore single-cell methylome and transcriptome to reveal the underlying transcriptional regulatory networks associated with epigenetic heterogeneity of ESCs.

## Author summary

DNA methylation is an epigenetic mark with covalent modification that occurs directly on genetic material. In vertebrates, the most common form of DNA methylation is 5-methylcytosine (5-mC) at which a methyl group (CH3) is attached to the cytosine nucleotide, especially in the context of CpG dinucleotide. DNA methylation has important regulatory roles in a broad range of biological processes and diseases, such as embryonic stem cells (ESCs) differentiation and development. ESC populations can be strikingly heterogeneous in DNA methylation. Emerging single-cell methods for capturing DNA methylation are being developed with the exciting potential to investigate the DNA methylation feature within complex and heterogeneous tissues. In this study, we implemented a computational pipeline to infer cell-subset specific methylation of ESCs from single-cell methylome. Through integrative analyses with transcription factor binding and single-cell transcriptome, we explored the underlying regulatory mechanisms associated with methylation heterogeneity in ESCs to interpret the biological functional relevance of methylation variations.

## Introduction

Embryonic stem cells (ESCs) have a wide range of applications in both basic research and pre-clinical drug screening. ESCs are characterized with the capacity to self-renew and to differentiate into multi-lineage cells [1, 2]. While continuously proliferating, the undifferentiated ESCs are heterogeneous cellular populations corresponding to various differentiation potentials [3, 4]. Growing evidence indicated that heterogeneous ESCs display substantial variations in gene expression [5], transcription factor regulation patterns [6, 7], and epigenetic modifications including DNA methylation [8]. The heterogeneous expression of transcription factors (TFs) is responsible for lineage specific differentiation [9] and may underlie the mechanism that allows ESCs to exit self-renewal cycle and enter into various differentiation paths [7]. The recruitment of TFs to their binding sites may depend on DNA methylation and thus the binding activities of some TFs are methylation-dependent [10]. On the other hand, TF binding may modulate chromatin configuration and contribute to the regulation of DNA methylation [11, 12]. Consequently, the interplays between TF binding and DNA methylation orchestrate gene expression. Despite these increased understandings, the connections among TF binding, DNA methylation, and gene expression in ESCs remain largely unexplored at the single-cell level.

During cell differentiation, dynamic DNA methylation changes occur and have been recognized as needs for lineage-specific expression of developmentally regulated genes [8, 13]. Regular bisulfite sequencing data sets derived from various tissues are informative to identify tissue specific DNA methylation. However, in tissues with a mixed cell population, each cell subset may have a distinct epigenetic landscape with a specific set of genomic loci differentially methylated. For experiments using bulk tissues, it remains challenging to determine the cell-to-cell methylation variation. With the advances in single-cell sequencing technologies, single-cell reduced representation bisulfite sequencing (scRRBS) [14] and single-cell bisulfite sequencing (scBS-seq) [15, 16] have been exploited to profile genome-scale DNA methylation. Substantial heterogeneous DNA methylation patterns were observed in mouse ESCs [15]. Unfortunately, neither scRRBS nor scBS-seq could distinguish the methylation variations within a cell from the ones between cells. Within a cell, methylation variations may result from the differences between two alleles, i.e. allele-specific DNA methylation (ASM), or between the two complementary strands within a DNA molecule, i.e. asymmetric methylation (AM). Mouse ASM loci have been surveyed in a genome-wide study with brain methylomes generated from reciprocal crosses between two distantly related inbred strains [17]. AM can be assessed with the hairpin bisulfite sequencing technique, which generates methylation data for two complementary DNA strands simultaneously [18]. To compare the methylomes derived from single cells, it is necessary to consider ASM and AM, the two types of methylation variations within a cell.

In this study, we implemented a pipeline to identify the epigenetic heterogeneity from scBS-seq datasets of mouse ESCs and explored the correlations among DNA methylation and gene expression. Using information from previous map of allele specific methylated loci [17] and the genome annotation of asymmetric methylation for mouse ESCs [18], we were able to propose a statistical approach called the “beta mixture model” to infer the genomic regions exhibiting cell subset-specific methylation (CSM) pattern. Furthermore, we integrated the methylomes and transcriptomes at the single cell level as well as the profiles of TF bindings enriched in the putative CSM loci identified to decipher the epigenetic heterogeneity of mouse ESCs.

## Results

### Methylation profiles of ASM and AM loci in single-cell methylomes derived from mouse ESCs

To assess DNA methylation variations within and across single cells, we started with the scBS-Seq data generated with the random priming method for nineteen mouse ESCs [15]. We first extracted genomic segments with four neighboring CpG dinucleotides in any given sequence read (**S1A Fig)**. From the nineteen methylomes, 2,875,509 distinct 4-CpG segments were obtained and the number of 4-CpG segments varied from 98,586 to 1,054,970 in the 19 cells (**S1A Fig & S1 Table**). The average read depth of those 4-CpG segments in each cell varied from 1.1 for segments identified in only one cell to 35 for the segments identified in all 19 cells (**S1B Fig**). Among the total 2,875,509 4-CpG segments, only 701 were present in all 19 cells and 917,687 were identified in at least five cells (**S1C Fig**). 93.3% of the 701 4-CpG segments were with ≥5Xs read coverage in the 19 cells on average (**S1D Fig**), and 79% of these 701 segments were distributed in 5’UTR compared to 13.3% for all 2,875,509 segments (**S1E Fig**). This indicates the biased distribution of sequence reads on genome for single cell methylomes.

**Fig 1:**
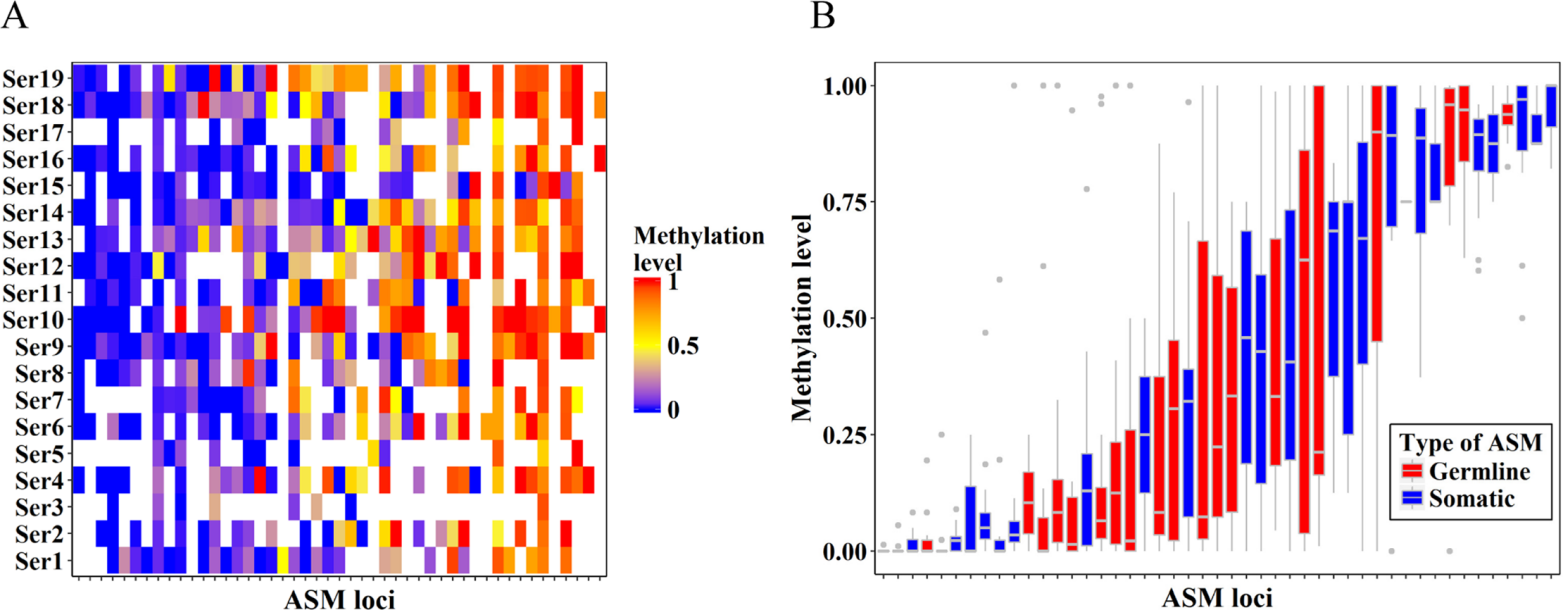
The methylation profile of ASM loci. (A) Heatmap of the methylation level of 47 ASM loci in 19 cells. The methylation levels were represented by color gradient from blue (unmethylation) to yellow (partial methylation) until to red (full methylation), with white color representing missing data of the locus in that cell. (B) Boxplot of the methylation level of 47 ASM loci across single cells, with germline and somatic ASM loci marked separately.

We next examined methylation levels for allelic specific methylated loci in single cell methylomes. Within a cell, theoretically, the methylation levels of ASM loci should be around 50%. Due to loss of DNA content during library preparation, PCR bias, and low sequence depth, the two alleles from a single cell may not present equally in sequencing data. To assess the representation of methylation patterns for two alleles, we examined the methylation profiles of ASM loci reported in a previous study [17]. These ASM loci were identified with brain tissues derived from reciprocal crosses between two distantly related mouse strains. We focused on the parent-of-origin dependent (imprinted) ASM at 1,952 CG dinucleotides in 55 discrete genomic loci, including 21 germline ASM loci which acquire allelic methylation status during gametogenesis and maintain throughout development, and 34 somatic ASM loci of which the allelic methylation states arise late in development in a tissue-specific manner [17]. The methylation levels for 47 of these ASM loci could be determined in at least one single-cell methylome (**S2 Table** and **Fig 1**). We calculated the methylation levels of these 47 ASM loci for each methylome and obtained 546 data points. Surprisingly, only 32 out of the total 546 data points are with methylation level between 0.4 and 0.6 (**Fig 1A**). In addition, the methylation levels of 47 ASM loci, including germline imprinted ones, are highly variable among single cells **(Figs 1B & S2A)**. Thus, the majority of ASM loci may be reported as cell subset-specific methylated: hyper-methylated in some cells while hypo-methylated in others.

We next examined the methylation profiles of asymmetric methylated loci in single-cell methylomes. In a DNA molecule, the CpG dyads on the two complementary DNA strands usually show highly symmetric methylation pattern [18–20]. However, strand-to-strand methylation variation has been observed in mouse ESCs. Using the hairpin bisulfite sequencing strategy, our recent study showed that approximately 12% of CpG dyads are asymmetrically methylated in undifferentiated ESCs [18]. In particular, 65.2% of half-methylated (methylation level at 50%) cytosines are due to asymmetric methylation. Apparently, CpG sites with intermediate methylation level may pose a challenge to the identification of CSM in single-cell methylomes, in particular for those with low sequence depth.

To explore CpG sites with asymmetrical methylation (AM), we integrated the hairpin bisulfite sequencing data and single-cell methylomes generated for mouse E14TG2a ESCs. From the hairpin methylome, we identified a total of 12,042 4-CpG segments as AM loci which have at least a pair of hairpin sequence reads showing one strand as completely methylated and the other strand as completely unmethylated. We further analyzed the single cell methylomes and identified 7,209 4-CpG segments as AM loci with both completely methylated and completely unmethylated reads within a single cell. We obtained 19,162 AM loci in total by merging the results from the hairpin bisulfite sequencing data and single cell methylomes. Similar to the observation made for ASM loci, the methylation levels of these 19,162 AM loci vary substantially across cells (**S2B and S2C Fig**).

### Beta mixture model to infer putative CSM loci

**Fig 2:**
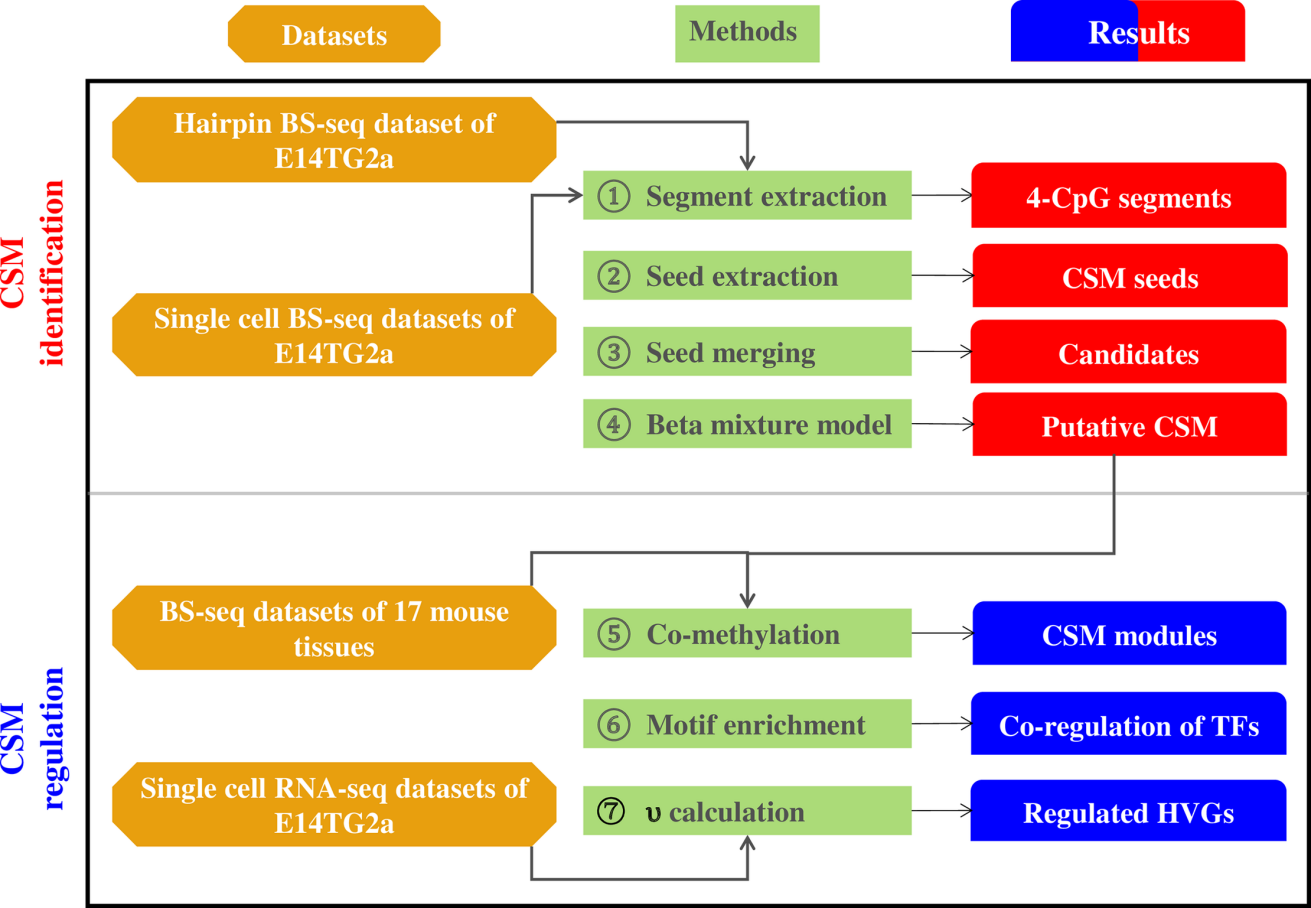
An overview of analysis pipeline to infer CSM with single-cell BS-seq dataset. Top panel illustrates the procedure of detecting putative CSM loci in mouse ES cells. Bottom panel illustrates the procedure of exploring the regulation mechanisms of putative CSM loci.

Since the two types of within-cell methylation variations, i.e. ASM and AM, may undermine the comparison of single-cell methylomes, we implemented a computational pipeline to assess the methylation heterogeneity among ESCs and infer putative CSM loci (**Fig 2**). The pipeline starts with the extraction of 4-CpG segments, excluding the known ASM and AM ones. We then defined CSM seeds as the 4-CpG segments that show complete methylated pattern in at least one methylome and complete unmethylated pattern in other methylomes. Overlapped CSM seeds were merged together to generate candidate CSM regions. Applying such a procedure to single cell methylomes, we obtained 7,161 candidate CSM regions covered by at least 5 cells and with at least 10 methylation counts within each candidate region in each cell.

Suppose that there are two methylation states: hyper-methylated and hypo-methylated in a given candidate region. However, the composition of each state is unknown. To decompose states, a beta mixture model is developed. Here we assumed that the methylation probabilities of hyper-methylated state and hypo-methylated state, denoted by θ^(1)^ and θ^(2)^, follow two distinct beta distributions. For each candidate region, the two probabilities were estimated by using the EM algorithms. One critical parameter is the methylation difference between two states for each candidate region, denoted by θ^(1)^—θ^(2)^. We conducted simulations to evaluate how the performance of our model is related to θ^(1)^—θ^(2)^ (**S3A & S3B Fig**). As shown in **S3A Fig,** the fraction of accurate prediction increased with the increasing of θ^(1)^—θ^(2)^ and became stable until θ^(1)^—θ^(2)^ reaching 0.3. Thus, for the beta mixture model, we determined the threshold of methylation difference between two methylation states as 0.3. We also checked the relationship between the estimated θ^(1)^—θ^(2)^ and real θ^(1)^—θ^(2)^ at different setting of λ which represented the proportion of the cells with hyper-methylated state in the given region, and found a high Pearson’s correlations, showing that the estimation of θ^(1)^—θ^(2)^ was accurate enough (**S3B Fig**).We further exploited the receiver operating characteristic (ROC) curve and the positive predictive value (PPV) to evaluate the model performance (**S3C & S3D Fig**). In the beta mixture model, we used Delta_min_ to represent the observed minimum methylation difference of the two methylation states. From the ROC curve, we found that the beta mixture model had high sensitivity and high specificity for different settings of Delta_min_ as well as high PPV for different settings of θ^(1)^—θ^(2)^. The false discovery rate (FDR) and false positive rate (FPR) decreased dramatically with the increase of θ^(1)^—θ^(2)^ until θ^(1)^—θ^(2)^ reached 0.3 (**S3E Fig**). In addition, to ensure the data quality, we required that a putative CSM loci should have data generated from at least 8 cells. With those parameters, 2,102 out of the total 7,142 candidate regions were inferred as putative CSM loci among ESCs.

### Putative CSM loci were characterized with the enrichment in CpG island (CGI) shelves and regions with histone marks for enhancer and promoter

**Fig 3:**
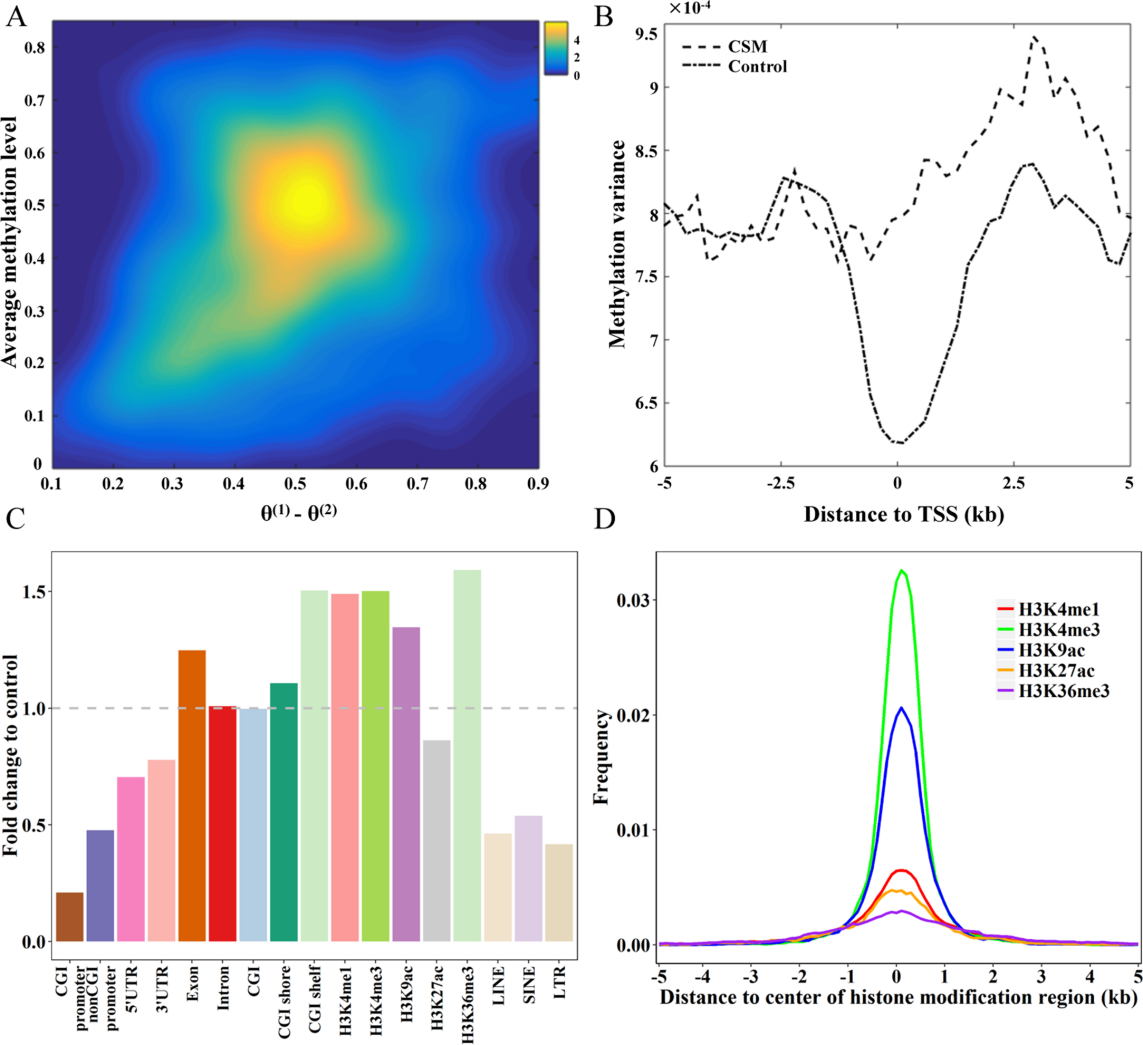
Characteristics of putative CSM loci. (A) Density scatterplot of θ^(1)^—θ^(2)^ (x-axis) and the average methylation level (y-axis) of putative CSM loci across 19 cells. The coloring indicates the density of putative CSM loci from low (blue) to high (yellow). (B) The methylation variance in putative CSM loci and control regions in 5kb flanking regions of TSS. (C) The fold change in the distribution of putative CSM loci across various genomic features compared to those of control regions. (D) The frequency of putative CSM loci distributed in the 5kb regions flanking the center of histone marks of H3K4me1, H3K4me3, H3K27ac, H3K9ac and H3K36me3.

We next assessed the methylation profiles, genomic characteristics, and DNA-related features of the 2,102 putative CSM loci (**Figs 3 & S4, S3 Tables**). We also produced our control region set including 46,642 regions by merging the 2,813,756 ASM-freed segments. Putative CSM loci are intermediated methylated with methylation levels centered around 50% across single cells (**Fig 3A**), while control regions tend to form two clusters, either hypermethylated or hypomethylated (**S4A Fig**). Additionally, the methylation differences between the two methylation states, i.e. θ^(1)^—θ^(2)^, are centered at 0.54 for putative CSM loci and 0.25 for control regions (**S4B Fig**). We calculated the methylation variance of putative CSM loci across cells and found that putative CSM loci exhibited significantly smaller methylation variance with average at 5.3e-04 compared to 5.7e-04 in control regions (Wilcoxon test, p value = 5.94e-09) (**S4B Fig**). By contrast, putative CSM loci exhibited higher methylation variance surrounding transcription start sites (TSSs) compared to control regions, especially in the downstream regions of TSSs (**Fig 3B**). In addition, we found that putative CSM loci were enriched in CGI shelves with a 1.5-fold increase compared with control regions, and 1.2-fold and 1.1-fold increase in exons and CGI shores, respectively (**Fig 3C**).

We further examined the correlation between DNA methylation and histone modifications. As shown in the **Fig 3C & 3D**, putative CSM loci show enrichment in regions with H3K4me1, H3K4me3, H3K9ac, and H3K36me3 marks, except H3K27ac. Since H3K4me1[21, 22], H3K4me3 [22, 23], and H3K9ac [24] are the histone marks for enhancers or promoters, while H3K36me3 marks indicate active transcribed genes and induce the DNA methylation of the gene bodies [25], this result suggests the regulation potential of the putative CSM loci on gene expression. Meanwhile, putative CSM loci are with higher GC content and CpG density (**S4C & S4D Fig**), which are known to be related to open chromatin and active transcription [26, 27]. In addition, compared to control regions, the sequences of putative CSM loci are more conservative among the placental mammals (**S4E Fig**).

### Co-methylation and co-regulation of putative CSM loci

**Fig 4:**
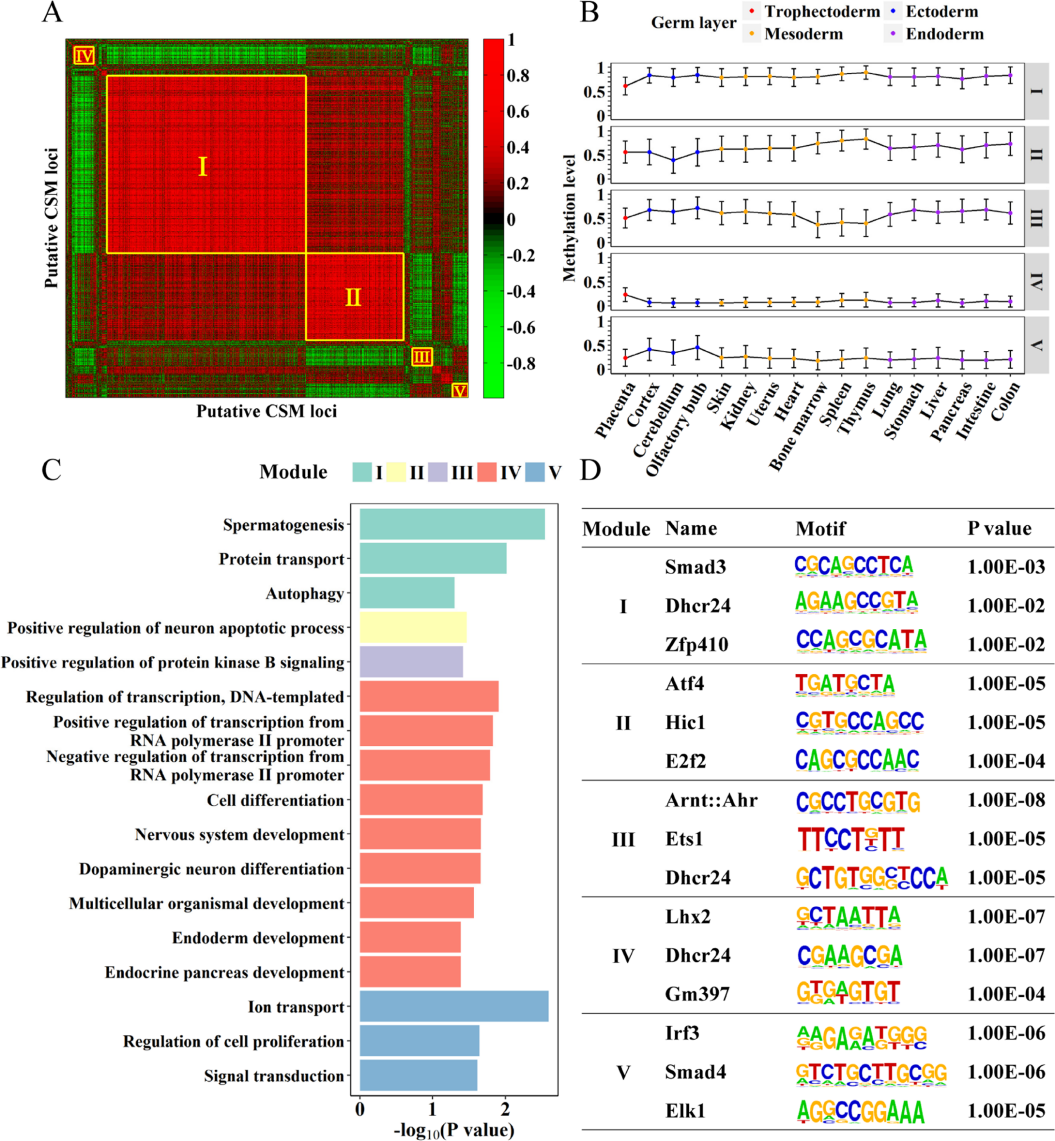
Co-methylation and co-regulation of putative CSM loci. (A) Heatmap of pair-wise Pearson’s correlations of putative CSM loci according to their methylation levels in 17 mouse tissues, with top five co-methylated modules marked. (B) The methylation profiles of the top five modules in the 17 mouse tissues, with circle showing the average methylation level, and the error bar showing the standard deviation. Tissues deriving from different germ layers are marked. (C) The significance of GO terms enriched for each module. P values were reported using NCBI DAVID annotation tool and scaled to–log10 based. (D) Top three TF motifs enriched in each module. P values were determined using Homer software.

To explore the association among the 2,102 putative CSM loci identified in mouse ESCs, we determined the methylation profiles of these loci in 17 mouse tissues spanning all three germ layers and extraembryonic placenta derived from trophectoderm [28] and performed co-methylation analysis to cluster these CSM loci into modules. For the 2,094 (99.7% of 2,102) putative CSM loci with data available in all 17 tissues, we calculated their pairwise Pearson's correlations in methylation level, and identified five major co-methylated modules (**Fig 4A**) which show distinct methylation profiles across different tissues (**Fig 4B**). An early differentiation event during embryonic development is the segregation of trophectoderm and inner cell mass [29]. Intriguingly, compared to those in other tissues, the methylation levels in placenta are lower for the putative CSM loci in module I but higher for those in module IV. Putative CSM loci in module II are hypomethylated in cerebellum (ectoderm-derived tissue) and putative CSM loci in module III are hypomethylated in bone marrow, spleen and thymus (blood-producing, mesoderm-derived tissues), while putative CSM loci in module V show higher methylation level in ectoderm-derived tissues.

To characterize the function of genes associated with co-methylated CSM modules, we determined genes with putative CSM loci located within [-10k, 2k] from TSS and then performed GO analysis using DAVID annotation tool [30, 31] to check the enrichment of GO terms for biological process (**Fig 4C**). For the largest module I, GO terms including protein transport and autophagy were identified to be significant. Autophagy is recognized to promote cell survival and involved in the development of human placenta [32, 33]. Genes in protein transport pathways are important for placenta function, since placenta plays an important role in the feto-maternal exchange processes via classic membranous transport mechanisms, i.e. the transportation capacity of the placenta. For module II and module III, the terms of neuron apoptotic process and positive regulation of protein kinase B signaling were identified, and were found to be related to the cell fate regulation during the development of cerebellum [34] and of hematopoietic lineages [35], respectively.

DNA methylation affects the bindings of transcription factors (TFs) to their targets [10], while TFs binding may prevent or facilitate the methylation on their binding sites [11, 12]. Hence, specific TFs could cooperate with DNA methylation to regulate gene expression. To examine whether co-methylated loci are under the control of the same set of TFs, we performed motif enrichment analysis with HOMER software [36] (**Fig 4D and S4 Table**). Intriguingly, each co-methylated module is associated with a distinct set of transcription factors, whose functions have been linked to the tissues associated with modules. More specifically, transcription factor *Dhcr24* was found to be the regulator for the putative CSM loci in both modules I and IV. The *Dhcr24* gene is involved in cellular lipid metabolism and cholesterol biosynthesis [37], and cholesterol is of vital importance for fetal development, thus the expression of *Dhcr24* in placenta would provide a means to satisfy the high requirement for cholesterol in fetus [38]. Downregulation of this gene was detected in intrauterine growth restriction placentas compared to normal placentas [39], which indicated that the decreased expression of this gene in the placenta influenced the cholesterol supply to the fetus, and contributed to the poor fetal growth. The enriched *Hic1* [40] in module II, and *Ets1* [41, 42] in module III were essential for normal development of cerebellar and for the establishment of differentiation potentialities of hematopoietic tissues in mesoderm layers, respectively.

### Putative CSM loci may underlie variable gene expression in ESC

To further investigate the role of DNA methylation in transcription regulation, we re-analyzed a single-cell RNAseq dataset derived from IB10 cell line [43], a sub-clone of E14 ESCs we analyzed for the single cell methylomes in this study. Following the procedure described in the previous study [43], we identified 2,266 highly variable genes (HVGs), which were genes with over-dispersed abundance compared to those transcripts with non-fluctuating expression in all cells and showed much higher υ statistics than other genes (**S5A Fig**) [43]. To explore how the CSM contributes to the variation in gene expression, we examined the HVGs in genes overlapped with putative CSM loci (**S5B Fig**). We determined genes with putative CSM loci localized in the distal upstream region ([-10k, 2k] of TSS), proximal upstream region ([-2k, 0.5k] of TSS), and gene body ([-10k of TSS, TES]). A total of 927 genes with their distal upstream regions overlapped with putative CSM loci showed significant enrichment in the list of HVGs, with 134 of these 927 genes highly variably expressed among ES cells (Chi square test, p value = 2.5e-02). In contrast, no significant overlap was observed among HVGs and genes with putative CSM loci in their proximal upstream regions or gene bodies (Chi square test, p value = 0.70 and 0.11, respectively). This result indicates that the methylation heterogeneity in distal upstream region might underlie the variable gene expression in ESCs rather than proximal upstream region or gene body.

We then examined the methylation differences of HVGs between two methylation states, i.e. θ^(1)^—θ^(2)^. Interestingly, for those genes with putative CSM loci in their gene bodies, we found that HVGs showed significantly higher θ^(1)^—θ^(2)^ than non-HVGs (Wilcoxon test, p value = 4.1e-02), while for genes with non-CSM loci in their gene bodies, the θ^(1)^—θ^(2)^ of HVGs were significantly lower than those of non-HVG (Wilcoxon test, p value = 4.1e-06) (**S5C Fig**). This indicates that other factors such as histone modifications may be involved in regulating genes lack of CSM, of which the expression variability showed independence to the methylation difference. Even for genes with putative CSM loci, the CSM are only partially responsible for the variable expression. This result is similar to a recent study which demonstrated that for genes with variably methylated promoters among single cells, about 26.1% of them are significantly correlated with gene transcription, while for genes with hypomethylated promoters, 51% of them exhibit dynamic expression across cells [44]. Altogether, these results suggest a complex regulating role of DNA methylation on gene expression, either in promoter or gene body.

## Discussion

Embryonic stem cells are characterized by high cellular heterogeneity and consist of various cell subsets that express different levels of specific markers (such as Stella, Nanog and GATA-6) and differ in bias toward self-renewal or differentiation [4]. Single cell “omics” studies provide data in an unprecedented resolution to achieve understandings of the cellular complexity in multicellular organisms. Currently, a few single cell methylome datasets are available [14–16] but how to analyze and interpret methylation variation among single cells is far from clear. For cells in multicellular organisms, the genomic DNA contents are nearly identical, if not the same. However, at the epigenome level, dynamic DNA methylation is key to diverse cellular functions. In this study, we proposed a computational pipeline to infer CSM with scBS-seq data derived from mouse ES cells. To our knowledge, this study is the first attempt to explore single cell methylomes for CSM in heterogeneous embryonic stem cells. The pipeline implemented in this study may also be applied to other emerging single-cell methylation data sets.

Single-cell methylomes are frequently with low read depth, which greatly limits the distinction of CSM from ASM and AM. Such a limitation has also been pointed out in a recent study. Hu et al. discovered a high rate of allele drop-out while applying single-cell techniques, resulting that the vast majority of assayed CpGs represent only one of two possible alleles [44]. To overcome such a limitation, the pipeline implemented in this study took the within-a-cell interference into account, and annotated ASM from previous knowledge and AM based on hairpin bisulfite sequencing data from our previous study and scBS-seq datasets. Current single-cell epigenetic studies primarily focused on measuring methylation heterogeneity by estimating the cell-to-cell methylation variance [44–46]. However, methylation variance may not reflect the heterogeneity attributed to different methylation states. For example, the higher methylation variance could be caused by methylation levels following continuous uniform distribution than bi-modal distributed ones, whereas the latter is more likely to be seen in a population of mixed cell subsets. In contrast to aforementioned studies [44–46], we model the methylation data on putative CSM loci with a beta mixture model. Based on this model, we determined the difference of the estimated methylation probabilities between the two methylation states and provided statistical justification to infer putative CSM loci. As a side note, it was found that, when divided into hyper-and hypo-methylated clusters, such putative CSM loci exhibited higher methylation difference (θ^(1)^—θ^(2)^) but smaller methylation variance (**S4C Fig**).

Our analysis pipeline for CSM inference accepts single cell methylomes and excludes genomic loci associated with allele-specific and asymmetric methylation. It has several limitations on the requirements of prior knowledge and data inputs. 1) We assume a majority of allele-specific methylated loci have been identified in previous studies [17]. However, it remains challenging to determine the genome loci associated with stochastic allele-specific methylation and the parental origins of the conservative genomic loci lacking of SNPs across mouse strains. Thus, the existing list of ASM loci may not be comprehensive. 2) Our recent studies [18, 47] on asymmetric DNA methylation suggest that fast replicating cells may have a large number of asymmetrically methylated CpG dyads while terminally differentiated cells have much fewer. Although asymmetric methylated CpG sites tend to be widely distributed [18], some clusters of asymmetric methylated CpG sites in stem cells may end up as a source of cell specific methylated loci if the methylation statuses of two DNA strands segregating into two daughter cells are stable during cell duplication. 3) Apparently, the determination of putative CSM loci is highly dependent on the data quality of single cell methylomes, in particular the number of single cell sequenced, the genome coverage and read depth for each methylome. Currently, only very limited number of methylomes were determined at the single cell level and with low genome coverage. This greatly limits the downstream methylome comparisons and co-methylation analysis of CSM clusters.

Despite all the aforementioned limitations, we were able to infer a number of putative CSM loci in mouse ESCs and made several interesting observations. The genome distribution analysis for putative CSM loci show that these loci are enriched in CGI shelves and genomic regions with histone marks for enhancer and promoter. We explored the methylation profiles of putative CSM loci in adult mouse tissues to perform co-methylation analysis. The co-methylation analysis provides valuable information for understanding on the biological readouts of epigenetic heterogeneity. Some putative CSM loci in co-methylated modules show placenta specific methylation profile. This suggests that, within a population of ESCs, some cells may be pre-marked at the epigenetic level and with the potential to differentiate into placenta tissue. In addition, TFs playing important roles in tissue specification were enriched in the co-methylation modules. More interestingly, the integration with single cell RNAseq data indicates that the putative CSM loci are associated with highly variable genes. The three-step procedure implemented in this study will provide lists of co-methylation modules, co-regulation of TFs, and underlying highly variable expression. Such a process paves the way to integrate “omics” data sets from multiple layers and to explore epigenetic regulation at a module-based level.

## Methods

### Analyses of scBS-seq datasets and hairpin BS-seq dataset

Methylomes of mouse ES cells (E14TG2a) were downloaded from Gene Expression Omnibus (GEO) database (GSE56879), including 19 scBS-seq datasets of cells cultured in serum/LIF [15] and one hairpin BS-seq dataset (GSE48229) [18]. Our scBS-seq data analysis followed the processing steps provided in Smallwood et al. 2014 [15]: 1) perform adaptor trimming with Trim Galore! (v0.3.7); 2) map reads to human genome (GRCh38/hg19) in pair-end mode to remove contaminated reads and then map the unmapped reads to mouse genome (GRCm38/mm10) in single-end mode using Bismark [48] (v0.7.7); 3) perform duplication removal using picard-tools (v1.118); 4) perform methylation calling with Bismark [48] (v0.7.7). For hairpin BS-seq dataset, HBS analyzer [49] was employed. For both scBS-seq data and hairpin BS-seq data, all segments with four neighboring CpG sites in any sequence read were extracted from autosomes. In this study, the methylation level was determined as the ratio of the methylated cytosine counts to the total cytosine counts.

### Annotation of ASM and AM loci

The genomic coordinates of mouse ASM loci and the annotation of either ‘germline’ or ‘somatic’ ASM were retrieved from a previous study [17], and were lifted to mm10 using liftOver. The AM loci were determined from hairpin BS-seq data [18] and scBS-seq data [15]. With hairpin BS-seq data, the AM loci were defined as 4-CpG segments with completely methylated pattern on one strand and completely unmethylated pattern on the other strand in a pair of hairpin sequence reads. For the scBS-seq dataset, the 4-CpG segments with at least one completely methylated read and one completely unmethylated read in one cell were defined as AM loci.

### Inference of candidate CSM regions

Three steps were taken to infer candidate CSM regions. 1) The determination of seeds for CSM: The 4-CpG segments overlapped with known ASM loci were filtered from the total segments. Bipolar methylated segments were selected from the remaining segments, which were defined as the ones with completely methylation in one single-cell methylome and completely unmethylation in any other single-cell methylome. After filtering out AM loci, the remaining bipolar methylated segments were defined as CSM seeds. 2) The extension of CSM seeds: Each CSM seed as well as other ASM-filtered segments were extended to include upstream and downstream 100 bp regions. Extended CSM seeds overlapped with other extended segments or seeds were merged into one, which ensured that each merged region included at least one CSM seed. 3) The extraction of candidate CSM regions: The merged regions covered by at least 5 single-cell methylomes and with at least 10 cytosine counts in each single-cell methylome were defined as candidate CSM regions. To produce a control set for putative CSM loci, all ASM-filtered segments were extended to include upstream and downstream 100 bp regions, merged with overlapped ones, and filtered with the same cutoffs of number of cells and cytosine counts as the candidate CSM regions.

### Empirical Bayesian estimation

Consider N single cells and R regions. For a given a region *r* from cell *i*(*r* = 1,2,…,*R*; *i* = 1,2,…,*N*), there are *c*_*ri*_ CpG sites. For each CpG site, we assume that the methylated count follows binomial distribution with a common methylation probability. We further assume that there are a total of *n*_*jri*_ read counts for the *j*th CpG site (*j* = 1,2,…,*c*_*ri*_). Then, on this CpG site, we have the methylated count

$$m_{jri}∼Binomial\;(n_{jri},\theta_{ri}).$$ (1)

Denote ${{M_{ri}^{\prime }=(m}_{1ri},m_{2ri},\ldots ,m_{c_{ri}ri})}^{T}$ and, ${{N_{ri}^{\prime }=(n}_{1ri},n_{2ri},\ldots ,n_{c_{ri}ri})}^{T}$ the joint probability function can be written as

$$f(M_{ri}^{\prime }|\theta_{ri};N_{ri}^{\prime })=\prod_{j=1}^{c_{ri}}C_{n_{jri}}^{m_{jri}}{{\theta_{ri}}^{m_{jri}}(1-\theta_{ri})}^{n_{jri}-m_{jri}}.$$ (2)

Since the true methylation probability *θ*_*ri*_ is unknown, we treat *θ*_*ri*_ as a random variable which follows beta distribution,

$$\theta_{ri}∼Beta(\alpha_{ri},\beta_{ri}).$$ (3)

By conjugacy, we have the posterior distribution of *θ*_*ri*_ that is also beta distribution

$$\mathrm{Pr}\left(\theta_{ri}|\alpha_{ri},\beta_{ri};M_{ri}^{\prime },N_{ri}^{\prime }\right)=Beta(\sum_{j=1}^{c_{ri}}m_{jri}+\alpha_{ri},\sum_{j=1}^{c_{ri}}n_{jri}-\sum_{j=1}^{c_{ri}}m_{jri}+\beta_{ri}).$$ (4)

The parameters of the prior distribution *α*_*ri*_ and *β*_*ri*_ are unknown. In order to estimate them, first, the beta distribution may be reparameterized by its mean *μ*_*ri*_ and precision *M*_*ri*_, that is

$$\mu_{ri}=\frac{\alpha_{ri}}{{\alpha_{ri}+\beta }_{ri}},\;M_{ri}=\alpha_{ri}+\beta_{ri}.$$

According to the previous assumptions of distributions, the marginal distribution of the methylated counts *m*_*jri*_ is then given by beta-binomial distribution. Second, the parameters *μ*_*ri*_ and *M*_*ri*_ of the beta-binomial distribution are estimated using an empirical Bayesian method [50]. Consequently, we obtain an estimation based on the method of moments

$${\hat{\mu }}_{ri}=\frac{\sum_{j}n_{jri}e_{jri}}{\sum_{j}n_{jri}},$$ (5)

where ${\hat{\mu }}_{ri}$ is the weighted mean of observed methylation level *e*_*jri*_, and $e_{jri}=\frac{m_{jri}}{n_{jri}},j=1,2,\ldots ,c_{ri}$. An estimation of precision *M*_*ri*_ may be obtained as

$${\hat{M}}_{ri}=\frac{{\hat{\mu }}_{ri}(1-{\hat{\mu }}_{ri})-s_{ri}^{2}}{s_{ri}^{2}-\frac{{\hat{\mu }}_{ri}(1-{\hat{\mu }}_{ri})}{N}\sum_{i=1}^{N}\frac{1}{\sum_{j=1}^{c_{ri}}n_{jri}}},$$ (6)

where $s_{ri}^{2}$ is the total weighted sampled variance

$$s_{ri}^{2}=\frac{\sum_{j}n_{jri}{\left(e_{jri}-{\hat{\mu }}_{ri}\right)}^{2}}{\sum_{j}n_{jri}}.$$

Based on (5) and (6), *α*_*ri*_ and *β*_*ri*_ are estimated as follows

$${\hat{\alpha }}_{ri}={\hat{\mu }}_{ri}{\hat{M}}_{ri},$$ (7)

$${\hat{\beta }}_{ri}=(1-{\hat{\mu }}_{ri}){\hat{M}}_{ri}.$$ (8)

In case that ${\hat{M}}_{ri}$ is negative [50], we assign ${\hat{\alpha }}_{ri}={\hat{\beta }}_{ri}=1$. In addition, for missing methylation data on some CpG sites for some cells, we set the two parameters of their methylated counts and total counts to zero.

### Methylation variance of cell to cell

To understand the methylation heterogeneity driven by CSM, we evaluate the methylation variance of cell to cell. To this end, we employ a random effect model to describe the variances across single cells. According to the posterior estimations of methylation probabilities above, we have the expectations and variances of the methylation probabilities of *θ*_*ri*_:

$${E(\theta }_{ri})=\frac{\sum_{j}m_{jri}+\alpha_{ri}}{\sum_{j}n_{jri}+\alpha_{ri}+\beta_{ri}},$$ (9)

$$var(\theta_{ri})=\frac{\left(\sum_{j}m_{jri}+\alpha_{ri})(\sum_{j}n_{jri}-\sum_{j}m_{jri}+\beta_{ri}\right)}{{\left(\sum_{j}n_{jri}+\alpha_{ri}+\beta_{ri}\right)}^{2}(\sum_{j}n_{jri}+\alpha_{ri}+\beta_{ri}+1)}.$$ (10)

Also, we assume that *μ*_*r*_ is the abstract methylation probability across single cells. Furthermore, $\Delta_{r}^{2}$ is defined as the variance of population; *δ*_*ri*_ is defined as the deviation from the average methylation probability across single cells; and *ε*_*ri*_ is a random effect. The observed methylation probabilities *θ*_*ri*_ with the corresponding variance *V*_*ri*_ for region *r* from cell *i* are considered to be a function of the abstract methylation probability *μ*_*r*_, *δ*_*ri*_ and *ε*_*ri*_:

$$\theta_{ri}=\mu_{r\theta }+\delta_{ri}+\varepsilon_{ri}.$$ (11)

To resolve the random effect model, a non-iteration algorithm was employed [51]. As a result, *μ*_*r*_ is estimated as a weighted mean of the observed methylation probabilities *θ*_*ri*_:

$${\hat{\mu }}_{r}=\frac{\sum_{i=1}^{N}w_{ri}^{*}\theta_{ri}}{\sum_{i=1}^{N}w_{ri}^{*}},$$ (12)

where

$$w_{ri}^{*}={\left(V_{ri}+{\hat{\Delta }}_{ri}^{2}\right)}^{-1}.$$ (13)

Also, the estimator of the methylation variance ${\hat{V}}_{r}$ is

$${\hat{V}}_{r}=\frac{1}{\sum_{i=1}^{N}w_{ri}^{*}},$$ (14)

where the 95% confidence interval of ${\hat{V}}_{r}$ is obtained from 1000 Bootstrap samplings.

### Clustering of single cell subpopulations

Suppose that there are *K* methylation states in a given region. As the composition of methylation state is unknown, a mixture model is employed to decompose the mixture methylation states. To this end, we focus on some candidate regions with methylation variation across cells. For a given region *r*, we assume that the proportion of the *k*th subgroup over the cell population is *λ*_*rk*_, where $\sum_{k=1}^{K}\lambda_{rk}=1$. As mentioned above, we assume that the number of methylated count for each CpG site in a given region follows binomial distribution and the methylation probability follows beta distribution. Then, we obtain the posterior distribution of methylation probability *θ*_*ri*_ in region *r* from cell *i*:

$$\mathrm{Pr}\left(\theta_{ri}|M_{ri}^{\prime },N_{ri}^{\prime }\right)=Beta(\sum_{j=1}^{c_{ri}}m_{jri}+\alpha_{ri},\sum_{j=1}^{c_{ri}}n_{jri}-\sum_{j=1}^{c_{ri}}m_{jri}+\beta_{ri}).$$

Since cells are grouped in the region, the methylation probabilities of the cells from a subgroup are assumed to be the same. Let $\theta_{r}^{\left(k\right)}$ denote the methylation probability of group *k*. Then, the probability for the observed methylation in cell *i* is:

$$\mathrm{Pr}\left(i\right)=\sum_{k=1}^{K}\mathrm{Pr}\left(k\right)*Pr(i|k)=\sum_{k=1}^{K}\lambda_{rk}\mathrm{Pr}\left(i|\theta_{r}^{\left(k\right)}\right).$$

According to the posterior distribution of methylation probability, the conditional probability of observing cell *i* from subgroup *k* is obtained:

$$\mathrm{Pr}\left(i|\theta_{r}^{\left(k\right)}\right)=\frac{\Gamma (\sum_{j=1}^{c_{ri}}m_{jri}+{\hat{\alpha }}_{ri})\Gamma (\sum_{j=1}^{c_{ri}}n_{jri}-\sum_{j=1}^{c_{ri}}m_{jri}+{\hat{\beta }}_{ri})}{\Gamma (\sum_{j=1}^{c_{ri}}n_{jri}+{\hat{\alpha }}_{ri}+{\hat{\beta }}_{ri})}{\left(\theta_{r}^{\left(k\right)}\right)}^{\sum_{j=1}^{c_{ri}}m_{jri}+{\hat{\alpha }}_{ri}-1}*{\left(1-\theta_{r}^{\left(k\right)}\right)}^{\sum_{j=1}^{c_{ri}}n_{jri}-\sum_{j=1}^{c_{ri}}m_{jri}+{\hat{\beta }}_{ri}-1},$$

where Γ(.) is the Gamma function.

Therefore, the joint likelihood function can be written as:

$$L(\Theta )=\prod_{i=1}^{N}\mathrm{Pr}\left(i\right),$$ (15)

where $\Theta ={\left({\lambda_{r1},\lambda_{r2},\ldots ,\lambda_{rK};\;\theta }_{r}^{\left(1\right)},\;\theta_{r}^{\left(2\right)},\ldots ,\theta_{r}^{\left(K\right)}\right)}^{T}$. The parameters Θ may be estimated by maximizing the log likelihood function:

$$\hat{\Theta }=arg\;\max_{\Theta }logL(\Theta )=arg\;\max_{\Theta }l(\Theta )=arg\;\max_{\Theta }\sum_{i=1}^{N}\log\mathrm{Pr}\left(i\right).$$ (16)

The optimized problem (16) may be resolved by the Expectation-Maximization (EM) algorithm by introducing a latent random variable *Y*_*i*_ which denotes the membership of cell *i*, that is *Y*_*i*_ = *k* if cell *i* is from subgroup *k*. Let Pr(*Y*_*i*_ = *k*) denote the probability of *Y*_*i*_ = *k*. Finally, we iteratively estimate all parameters based on the EM algorithm:

E-step:

$$Pr(Y_{i}=k|i,\Theta )=\frac{\lambda_{rk}\mathrm{Pr}\left(i|\theta_{r}^{\left(k\right)}\right)}{\sum_{k=1}^{K}\lambda_{rk}\mathrm{Pr}\left(i|\theta_{r}^{\left(k\right)}\right)},$$ (17)

M-step:

$$\left\{ \begin{array}{c} \lambda_{rk}=\frac{\sum_{i=1}^{N}Pr(Y_{i}=k|i,\;\Theta )}{N}\\ \theta_{r}^{\left(k\right)}=\frac{\sum_{i=1}^{N}Pr(Y_{i}=k|i,\;\Theta )(\sum_{j=1}^{c_{ri}}m_{jri}+{\hat{\alpha }}_{ri}-1)}{\sum_{i=1}^{N}Pr(Y_{i}=k|i,\;\Theta )(\sum_{j=1}^{c_{ri}}n_{jri}+{\hat{\alpha }}_{ri}+{\hat{\beta }}_{ri}-2)} \end{array}\right.,$$ (18)

here *k* = 1,2,…*K*,

where Pr(*Y*_*i*_ = *k*|*i*,Θ) is the posterior estimation of the probability of *Y*_*i*_ = *k* given the observed cell *i* and parameters Θ. In this study, we only focused on the bimodal methylation states by assuming a two-state model, that is *K* = 2.

### Assessment of beta mixture model

For each candidate region in a given cell, we considered two models: one is the beta mixture model; the other is a null model where only one cluster exists. We used likelihood ratio test to evaluate the goodness-of-fit of the two models to the data. The p-values are then adjusted by the Benjamini–Hochberg procedure [52]. In addition, we introduced a latent membership probability estimated by the beta mixture model to determine which cluster each single cell originates from in a given region, that is, the single cell *i* is from the first state if Pr(*Y*_*i*_ = 1) ≥ Pr(*Y*_*i*_ = 2), and from the second state otherwise. Besides, larger θ^(1)^—θ^(2)^ will lead to the more accurate estimation of the two states. We determined the cutoff of θ^(1)^—θ^(2)^ to be 0.3 based on simulation data. In the study, the regions with significant adjusted p-values and with θ^(1)^—θ^(2)^ (that is tuning parameter) greater than a given value were considered as putative CSM loci. Lastly, false discovery rate (FDR), true positive rate (TPR), false positive rate (FPR) and positive predictive value (PPV) are calculated for these putative CSM loci. A full description of the beta mixture model is provided in the S1 Text. The code and test data of the beta mixture model are available in the S1 Appendix and freely downloadable from https://github.com/Evan-Evans/Beta-Mixture-Model.

### Simulation

In the simulation study, we consider two cell subpopulations with distinct methylation probabilities. To evaluate the robustness of parameters estimation in the statistical model, we simulate data by setting the number of reads for each CpG site, the number of cells, and the rate of missing data. More specifically, the parameter λ is randomly sampled from unif[0, 1]; the read counts for each CpG site are sampled from a Poisson distribution with a prespecified mean that is considered as the read depth; and the methylated counts for each CpG site are sampled from binomial distribution with fixed methylation probabilities (i.e. $\theta_{r}^{\left(k\right)},\;k=1,2$) sampled from unif[0, 1]. We consider the estimated parameter to be accurate if the difference between the estimated value and the real value we set is less than 1e-2. All simulations are based on 10,000 independent samplings.

### Genomic features extraction

Genomic features were obtained from the UCSC Genome database [53], including annotations for gene structure (Refseq genes), CpG islands (cpgIslandExt), repetitive elements (RepeatMasker), and placental mammal conservation scores (phastCons60wayPlacental) in mm10. Promoters were defined as 1kb regions in the upstream of transcription starting sites (TSSs). CGI shores (2kb regions directly upstream and downstream of CpG islands) and CGI shelves (neighboring regions outwards from a CpG island shore and up to 4kb away from the CpG islands) were defined according to each CpG island. The information for DNA-related attributes including GC content, CpG density (defined as CpG observed vs. expected ratio) were extracted from the sequences of putative CSM loci. The histone modifications H3K27ac, H3K36me3, H3K4me1, H3K4me3, and H3K9ac for E14 cell line were obtained from the ENCODE Project [54] and lifted to mm10. Each histone peak was divided into 100 equal sized bins, and the frequency of putative CSM loci for each bin was calculated for plotting.

### CSM co-methylation and co-regulation analyses

We made use of 17 mouse tissue methylomes derived from a single pregnant female mouse (GSE42836), with an average depth of 8.2-fold genomic coverage per tissue, covering on average 79.7% of the CpG dinucleotides in the mouse genome [28]. The putative CSM loci with no methylation data available were filtered out. The methylation levels for the remaining 2,096 putative CSM loci (account for 99.6% of the total) were determined in each tissue. Pearson’s correlations were then calculated based on the methylation levels of each pair of putative CSM loci and further used for hierarchical clustering to determine co-methylation modules, with a correlation coefficient cut-off set as 0.75. The motif enrichment analyses were performed for each co-methylated module using Hypergeometric Optimization of Motif Enrichment (HOMER) [36].

### Single-cell RNAseq analysis

Single-cell RNAseq data for 933 cells derived from mouse IB10 cell line subcloned from E14 ESCs [43] were re-analyzed in this study. The expression profiles of these cells were downloaded from GEO (GSE65525). We referred to the filtering steps for genes in Zeisel et al. 2015 [55] to select genes with strong correlations with many others. First, genes with less than 10 UMI counts across the 933 mouse ESCs were removed (resulted in 23943 genes). Second, we calculated the Pearson’s correlation between each two genes based on their expression profiles across single cells. Next, a threshold of correlation among genes was set according to the 90th percentile of all the Pearson’s correlations (ρ = 0.166). We removed the gene if among the correlations involving this gene, only 4 or fewer correlation values were found to be larger than the threshold (resulted in 22660 genes). Lastly, the statistic score (υ) defined in Eq. (S13) in Klein et al. 2015 [43] was calculated and the genes with the top 10% υ were determined as HVGs (resulted in 2266 genes).

## Supporting information

S1 Fig Characterization of scBS-seq libraries. (A) Overview of the extraction of 4-CpG segments across 19 single cells. Two example segments composited by CpG 1~4, and CpG 2~5 were shown. Sequence reads derived from different cells were marked by different colors. Methylated and unmethylated patterns of each CpG were distinguished by black and white circles, respectively. (B) Average read depth of segments covered by different number of cells. (C) Number of segments covered by different number of cells. (D) The number of segments with read depth of 1X, 2X, 3X, 4X, and > = 5X covered by different number of cells. 19 ES cells are shown in x axis. Segments covered by different number of cells are shown in 19 facets, denoted as “#Cells: number”. (E) The frequency of segments covered by different number of cells in different genomic features.
(TIF)

S2 Fig The methylation profile of ASM and AM loci. (A) The distribution of range of methylation level (maximum methylation level–minimum methylation level) versus the average methylation level of each ASM locus across single cells. Each point represents one ASM locus, with germline and somatic ASM loci marked separately. (B) Heatmap of methylation level of 12,042 AM loci in 19 cells. The methylation levels are represented by color gradient from blue (unmethylation) to yellow (partial methylation) until to red (full methylation), with white color representing missing data of the locus in that cell. (C) Density scatterplot of the range of methylation level (maximum methylation level–minimum methylation level) versus the average methylation level of AM loci across single cells. Coloring indicates density of AM loci from high (black) to low (white).
(TIF)

S3 Fig Assessment of beta mixture model. (A) The distribution of the fraction of accurate prediction of the beta mixture model with different θ^(1)^—θ^(2)^ based on simulation data. Different settings of λ were shown in different colors. (B) Scatterplot of the estimated θ^(1)^—θ^(2)^ versus real θ^(1)^—θ^(2)^ based on simulation data. Different setting of λ were shown in different facets. (C) ROC curve of beta mixture model at different setting of Delta_min_. (D) PPV of beta mixture model at different setting of θ^(1)^—θ^(2)^. (E) Performance of beta mixture model with the θ^(1)^—θ^(2)^. The solid black line denotes the number of CSM. The solid red line represents the percent of false discovery rate (FDR). The solid blue line is the number of false positive CSM.
(TIF)

S4 Fig Characterization of putative CSM loci. (A) Density scatterplot of θ^(1)^—θ^(2)^ (x-axis) versus average methylation level (y-axis) in control regions across 19 cells. Coloring indicates density of control regions from low (blue) to high (yellow). (B) Violin plot of methylation variance, average methylation level, and θ^(1)^—θ^(2)^ of putative CSM loci across genomic features. Black dots mark the mean value; Black vertical lines indicate the standard deviation. Grey dash line marks the mean value of methylation variance, average methylation level, and θ^(1)^—θ^(2)^ of control regions. The distribution of (C) GC-content, (D) CpG density, and (E) placental mammal conservation of putative CSM loci and control regions.
(TIF)

S5 Fig Genes with putative CSM loci and highly variable genes of single ES cell transcriptome. (A) The υ statistics of HVGs and non-HVGs in log10 scale. (B) The number of HVGs and non-HVGs with putative CSM loci and non-CSM loci localized in their distal upstream region ([-10k, 2k] of TSS), proximal upstream region ([-2k, 0.5k] of TSS), and gene body ([-10k of TSS, TES]). P values are calculated by chi square test. (C) Distribution of θ^(1)^—θ^(2)^ of HVGs and non-HVGs with putative CSM loci and non-CSM loci localized in the gene body ([-10k of TSS, TES]). P values are calculated by wilcoxon rank sum test.
(TIF)

S1 Table Mapping details for 19 scBS-seq libraries. (XLSX)

S2 Table Annotation of coordinates of ASM loci in mm10 version. (XLSX)

S3 Table Statistical test for distribution of genomic features of putative CSM loci. (XLSX)

S4 Table Enrichment of TF binding motifs in putative CSM loci in five modules. (XLSX)

S1 Text A full description of beta mixture model. (DOCX)

S1 Appendix Beta mixture model and test data. (ZIP)
