# Supplementary figures and images for: Integrative single-cell omics analyses reveal epigenetic heterogeneity in mouse embryonic stem cells
Source: PLoS Comput Biol. 2018 Mar 21;14(3):e1006034. doi: 10.1371/journal.pcbi.1006034 (PMC5862410; doi:10.1371/journal.pcbi.1006034)

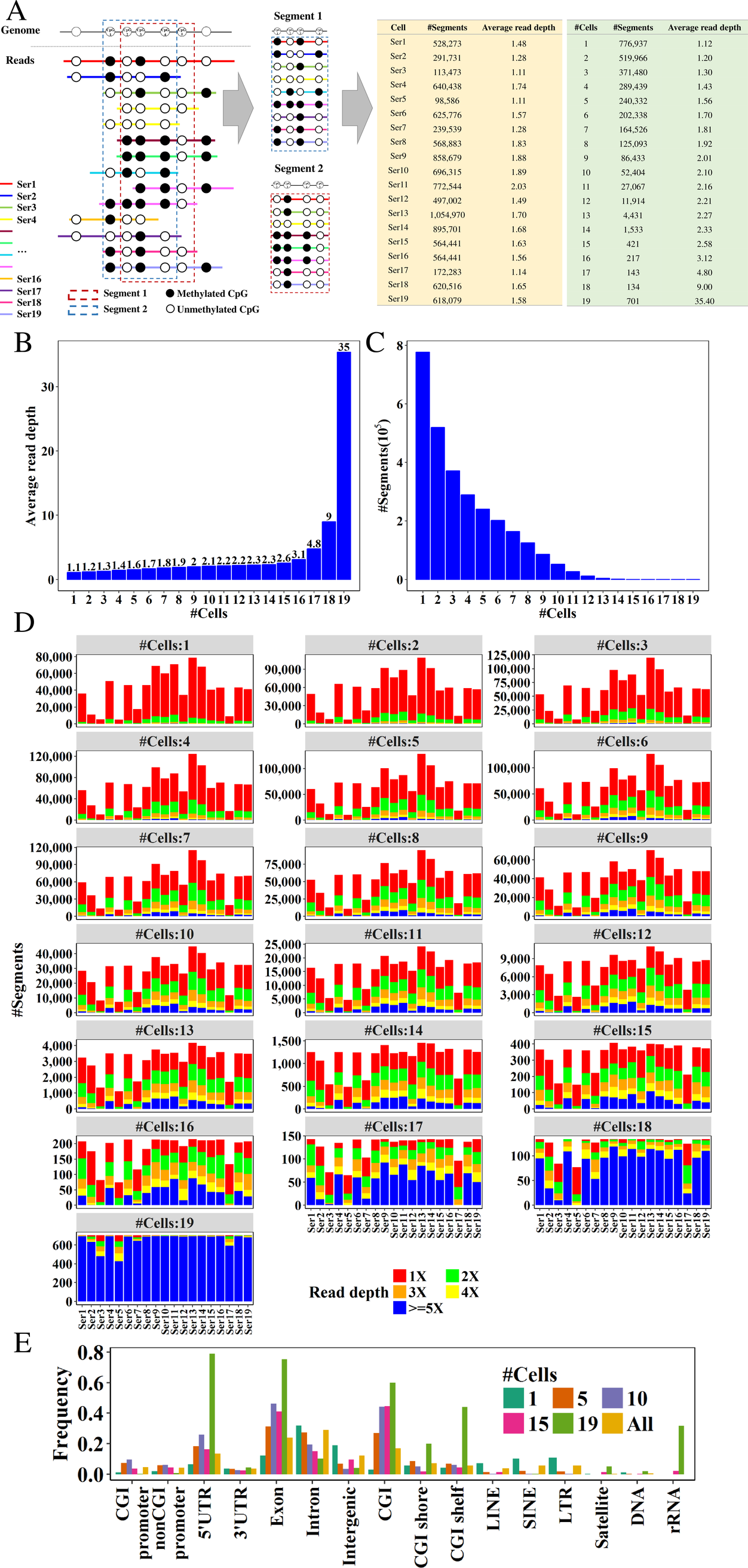

Supplement: S1 Fig — (A) Overview of the extraction of 4-CpG segments across 19 single cells. Two example segments composited by CpG 1~4, and CpG 2~5 were shown. Sequence reads derived from different cells were marked by different colors. Methylated and unmethylated patterns of each CpG were distinguished by black and white circles, respectively. (B) Average read depth of segments covered by different number of cells. (C) Number of segments covered by different number of cells. (D) The number of segments with read depth of 1X, 2X, 3X, 4X, and > = 5X covered by different number of cells. 19 ES cells are shown in x axis. Segments covered by different number of cells are shown in 19 facets, denoted as “#Cells: number”. (E) The frequency of segments covered by different number of cells in different genomic features. (TIF) [file pcbi.1006034.s001.tif]

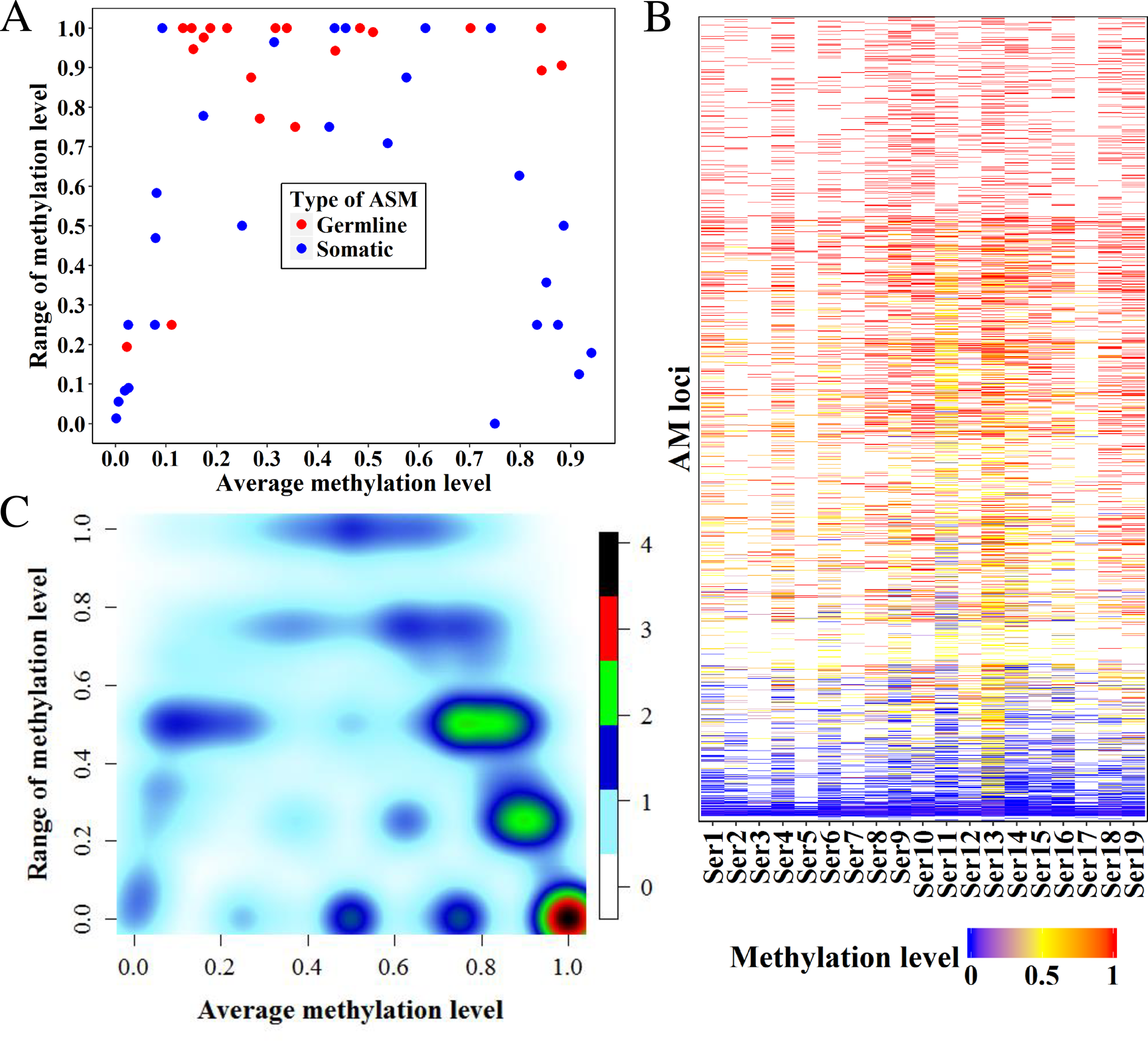

Supplement: S2 Fig — (A) The distribution of range of methylation level (maximum methylation level–minimum methylation level) versus the average methylation level of each ASM locus across single cells. Each point represents one ASM locus, with germline and somatic ASM loci marked separately. (B) Heatmap of methylation level of 12,042 AM loci in 19 cells. The methylation levels are represented by color gradient from blue (unmethylation) to yellow (partial methylation) until to red (full methylation), with white color representing missing data of the locus in that cell. (C) Density scatterplot of the range of methylation level (maximum methylation level–minimum methylation level) versus the average methylation level of AM loci across single cells. Coloring indicates density of AM loci from high (black) to low (white). (TIF) [file pcbi.1006034.s002.tif]

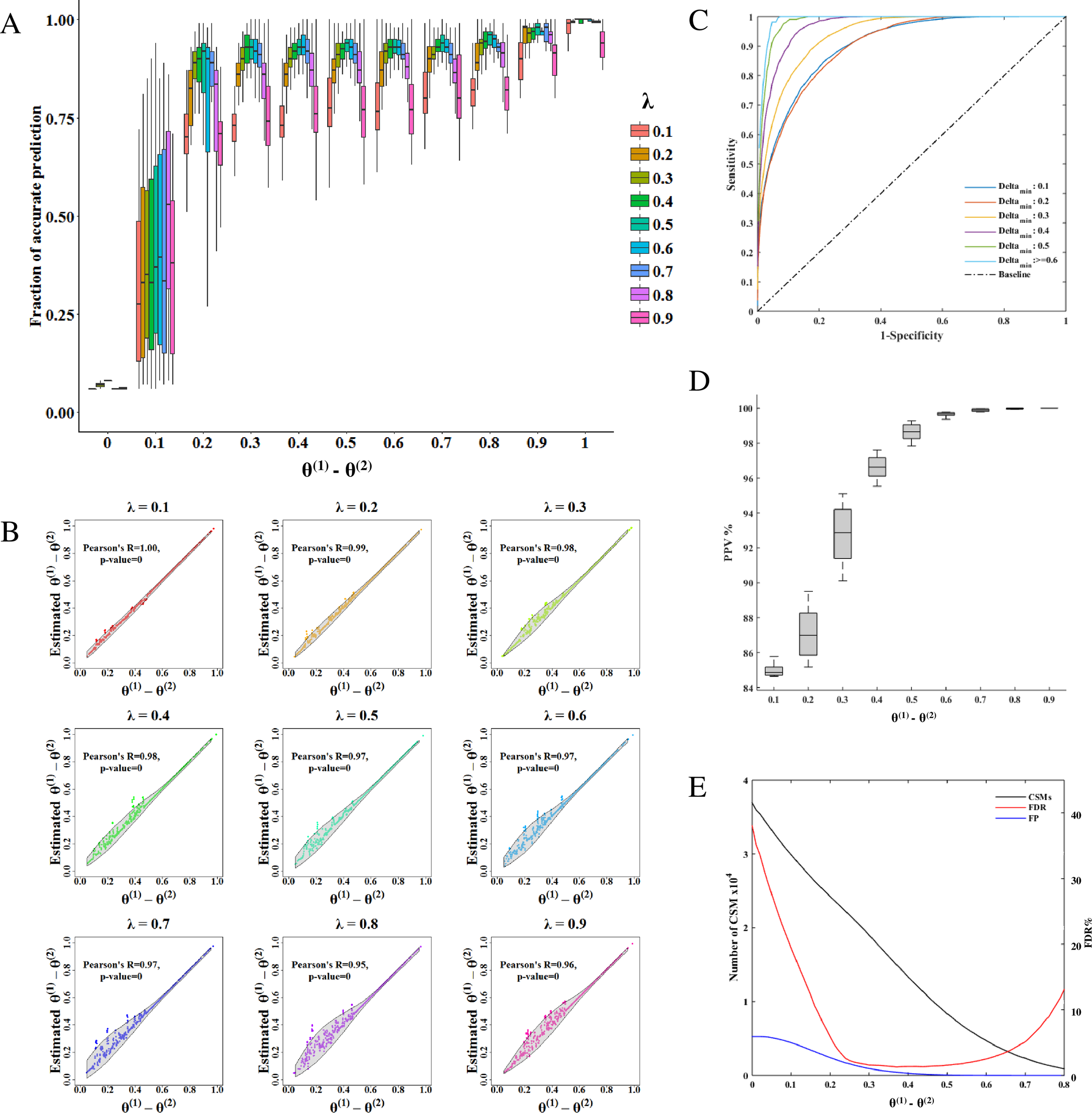

Supplement: S3 Fig — (A) The distribution of the fraction of accurate prediction of the beta mixture model with different θ(1)—θ(2) based on simulation data. Different settings of λ were shown in different colors. (B) Scatterplot of the estimated θ(1)—θ(2) versus real θ(1)—θ(2) based on simulation data. Different setting of λ were shown in different facets. (C) ROC curve of beta mixture model at different setting of Deltamin. (D) PPV of beta mixture model at different setting of θ(1)—θ(2). (E) Performance of beta mixture model with the θ(1)—θ(2). The solid black line denotes the number of CSM. The solid red line represents the percent of false discovery rate (FDR). The solid blue line is the number of false positive CSM. (TIF) [file pcbi.1006034.s003.tif]

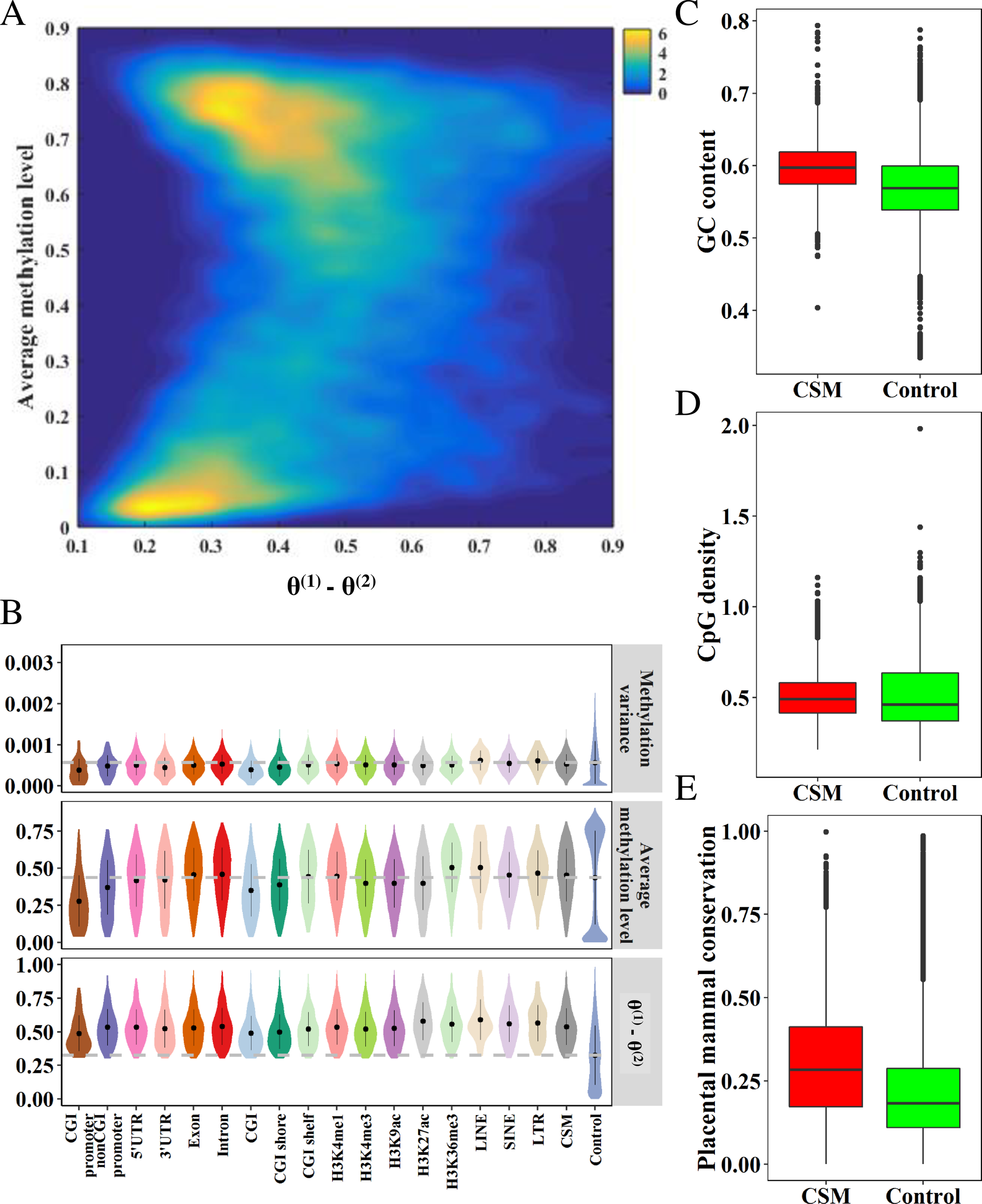

Supplement: S4 Fig — (A) Density scatterplot of θ(1)—θ(2) (x-axis) versus average methylation level (y-axis) in control regions across 19 cells. Coloring indicates density of control regions from low (blue) to high (yellow). (B) Violin plot of methylation variance, average methylation level, and θ(1)—θ(2) of putative CSM loci across genomic features. Black dots mark the mean value; Black vertical lines indicate the standard deviation. Grey dash line marks the mean value of methylation variance, average methylation level, and θ(1)—θ(2) of control regions. The distribution of (C) GC-content, (D) CpG density, and (E) placental mammal conservation of putative CSM loci and control regions. (TIF) [file pcbi.1006034.s004.tif]

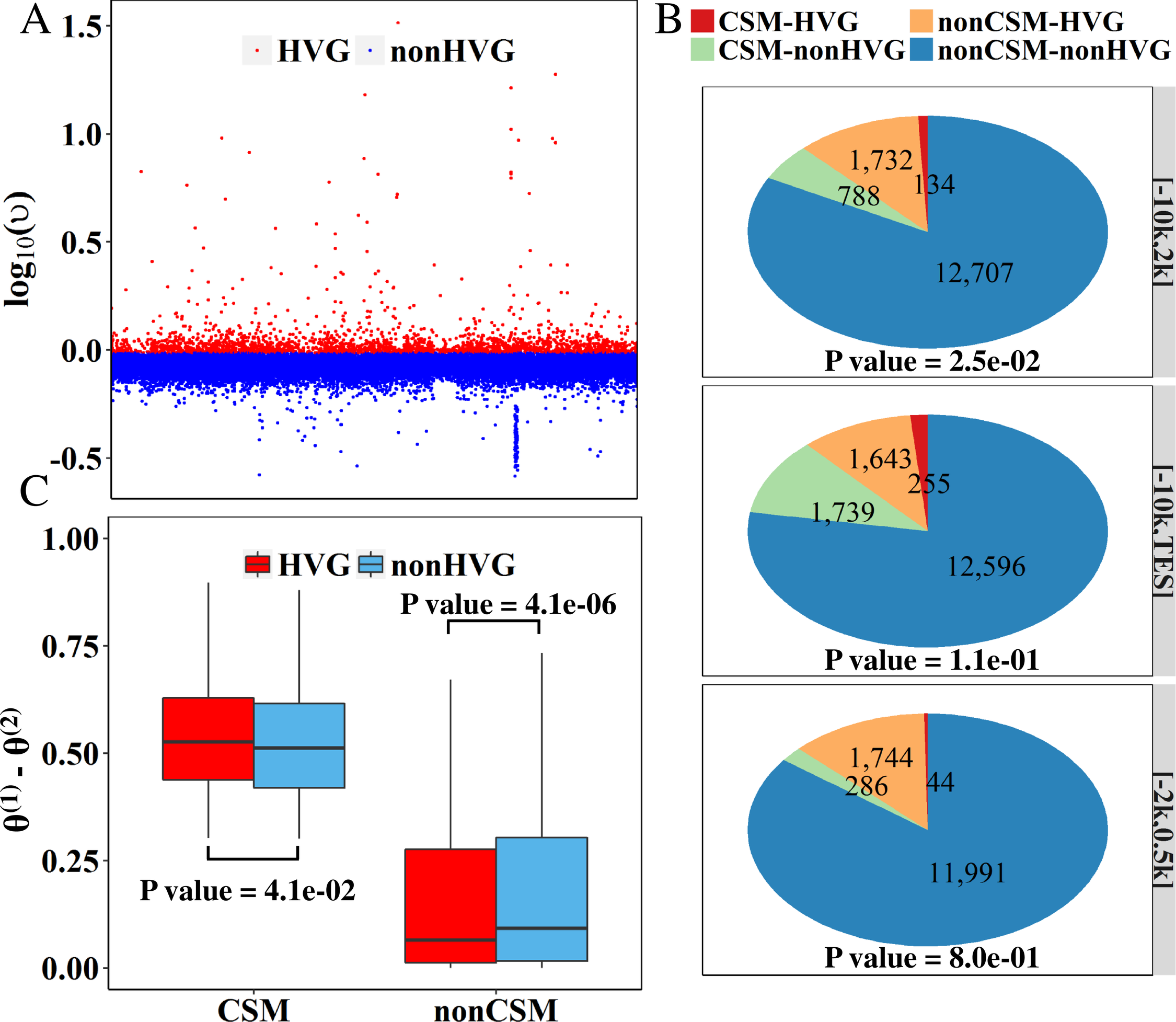

Supplement: S5 Fig — (A) The υ statistics of HVGs and non-HVGs in log10 scale. (B) The number of HVGs and non-HVGs with putative CSM loci and non-CSM loci localized in their distal upstream region ([-10k, 2k] of TSS), proximal upstream region ([-2k, 0.5k] of TSS), and gene body ([-10k of TSS, TES]). P values are calculated by chi square test. (C) Distribution of θ(1)—θ(2) of HVGs and non-HVGs with putative CSM loci and non-CSM loci localized in the gene body ([-10k of TSS, TES]). P values are calculated by wilcoxon rank sum test. (TIF) [file pcbi.1006034.s005.tif]
