# Supplementary material for: Integrative single-cell omics analyses reveal epigenetic heterogeneity in mouse embryonic stem cells
Source: PLoS Comput Biol. 2018 Mar 21;14(3):e1006034. doi: 10.1371/journal.pcbi.1006034 (PMC5862410; doi:10.1371/journal.pcbi.1006034)
Supplement: S1 Text — (DOCX) [file pcbi.1006034.s010.docx]

**Beta mixture model**

1. **Empirical Bayesian estimation**

For a given a region $r$from cell$i (r=1,2,\ldots,R; i=1,2,\ldots,N)$, there are $c_{ri}$CpG sites. For each CpG site, we assume that the methylated count follows binomial distribution with a common methylation probability. We further assume that there are a total of$n_{jri}$read counts for the $j\mathrm{th}$CpG site ($j=1,2,\ldots,c_{ri}$). Then, on this CpG site, we have the methylated count

$m_{jri}\boldsymbol{\sim}Bin(n_{jri},\theta_{ri})$. (1)

Denote ${{M_{ri}^{'}=(m}_{1ri},m_{2ri},\ldots,m_{c_{ri}ri})}^{T}\mathrm{and}{{N_{ri}^{'}=(n}_{1ri},n_{2ri},\ldots,n_{c_{ri}ri})}^{T}$, the joint probability function can be written as

$f\left( M_{ri}^{'} | \theta_{ri};N_{ri}^{'} \right)=\prod_{j=1}^{c_{ri}} C_{n_{jri}}^{m_{jri}}{{\theta_{ri}}^{m_{jri}}(1-\theta_{ri})}^{n_{jri}-m_{jri}}$. (2)

Since the true methylation probability $\theta_{ri}$ is unknown, we treat $\theta_{ri}$ as a random variable to follow beta distribution,

$\theta_{ri}\boldsymbol{\sim}Beta\left( \alpha_{ri},\beta_{ri} \right)$, (3)

Therefore, we have the posterior distribution of $\theta_{ri}$ that is also Beta distribution

$\Pr\left( \theta_{ri} | M_{ri}^{'},N_{ri}^{'} \right)=Beta\left( \sum_{j=1}^{c_{ri}} m_{jri}+\alpha_{ri},\sum_{j=1}^{c_{ri}} n_{jri}-\sum_{j=1}^{c_{ri}} m_{jri}+\beta_{ri} \right)$. (4)

The parameters of the prior distribution $\alpha_{ri}\mathrm{and}\beta_{ri}$are unknown. In order to estimate them, first, the beta distribution may be reparameterized by its mean $\mu_{ri}$and precision$M_{ri}$, that is

$\mu_{ri}=\frac{\alpha_{ri}}{{\alpha_{ri}+\beta}_{ri}} , M_{ri}=\alpha_{ri}+\beta_{ri}$.

According to the previous assumptions of distributions, the marginal distribution of methylated counts $m_{jri}$is then given by Beta-binomial distribution. Second, the parameters $\mu_{ri}$ and $M_{ri}$ of the beta-binomial distribution are estimated uing an empirical Bayesian method [[1](#_ENREF_1)]. Consequently, we obtain an estimation based on the method of moments:

$\hat{\mu}_{ri}=\frac{\sum_{j} n_{jri}e_{jri}}{\sum_{j} n_{jri}}$, (5) here $e_{jri}=\frac{m_{jri}}{n_{jri}}, j=1,2,\ldots,c_{ri}$

which is the weighted mean of observed methylation level $e_{jri}$. An estimation of precision$M_{ri}$may be obtained via [[2](#_ENREF_2)]:

$\hat{M}_{ri}=\frac{\hat{\mu}_{ri}\left( 1-\hat{\mu}_{ri} \right)-s_{ri}^{2}}{s_{ri}^{2}-\frac{\hat{\mu}_{ri}\left( 1-\hat{\mu}_{ri} \right)}{N}\sum_{i=1}^{N} \frac{1}{\sum_{j=1}^{c_{ri}} n_{jri}}}$. (6)

The total weighted sampled variance $s_{ri}^{2}$ is

$s_{ri}^{2}=\frac{\sum_{j} n_{jri}{(e_{jri}-\hat{\mu}_{ri})}^{2}}{\sum_{j} n_{jri}}$.

Therefore, based on (5) and (6), $\alpha_{ri}$and$\beta_{ri}$are estimated as follows

$\hat{\alpha}_{ri}=\hat{\mu}_{ri}\hat{M}_{ri}$, (7)

$\hat{\beta}_{ri}=(1-\hat{\mu}_{ri})\hat{M}_{ri}$. (8)

It is worth noting that because$\hat{M}_{ri}$can be negative, we assign$\hat{\alpha}_{ri}=\hat{\beta}_{ri}=1$in this case [[1](#_ENREF_1)]. In addition, for CpG sites with missing methylation data, we set their methylated counts and total counts as zeros.

1. **Methylation variance of cell to cell**

To understand methylation heterogeneity driven by cell subset specific methylation, we evaluate the cell-to-cell methylation variance. To this end, we employ random effect model to describe the variances across single cells. According to the posterior estimations of methylation probabilities above, we have the expectations and variances of methylation probabilities of $\theta_{ri}:$

${E(\theta}_{ri})=\frac{\sum_{j} m_{jri}+\alpha_{ri}}{\sum_{j} n_{jri}+\alpha_{ri}+\beta_{ri}}$, (9)

$\mathrm{var}\left( \theta_{ri} \right)=\frac{(\sum_{j} m_{jri}+\alpha_{ri})(\sum_{j} n_{jri}-\sum_{j} m_{jri}+\beta_{ri})}{\left( \sum_{j} n_{jri}+\alpha_{ri}+\beta_{ri} \right)^{2}(\sum_{j} n_{jri}+\alpha_{ri}+\beta_{ri}+1)}$. (10)

Also, we assume that $\mu_{r}$is the abstract methylation probability across single cells. Furthermore, $\Delta_{r}^{2}$is defined as the variance of population; $\delta_{ri}$is defined as the deviation from the average methylation probability across single cells; as well as $\varepsilon_{ri}$ is a random effect. The observed methylation probabilities $\theta_{ri}$ with the corresponding variance $V_{ri}$for the region $r$from cell$i$ are considered to be a function of the abstract methylation probability$\mu_{r}$, $\delta_{ri}$and $\varepsilon_{ri}:$

$\theta_{ri}=\mu_{r}+\delta_{ri}+\varepsilon_{ri}$, (11)

To resolve the random effect model, based on a non-iteration algorithm [[2](#_ENREF_2)], we estimate$\Delta_{r}^{2}$as follows

$\hat{\Delta}_{r}^{2}=max\{0,\frac{\sum_{i} w_{ri}{\theta_{ri}}^{2}-({{\sum_{i} w_{ri}\theta_{ri})}^{2}}/{\sum_{i} w_{ri}}-(N-1)}{\sum_{i} w_{ri}-({\sum_{i} {w_{ri}}^{2}}/{\sum_{i} w_{ri}})}\}$, (12)

In which $N$ represents the number of cells and $w_{ri}$is defined

$w_{ri}=\frac{1}{V_{ri}}$, (13) here$V_{ri}=var\left( \theta_{ri} \right)$.

Therefore, $\mu_{r}$is estimated as a weighted mean of the observed methylation probabilities$\theta_{ri}:$

$\hat{\mu}_{r}=\frac{\sum_{i=1}^{N} w_{ri}^{*}\theta_{ri}}{\sum_{i=1}^{N} w_{ri}^{*}}$, (14)

where$w_{ri}^{*}$is defined as$w_{ri}^{*}={(V_{ri}+\hat{\Delta}_{r}^{2})}^{-1}$(15). Also, the estimator of the cell-to-cell methylation variance $\hat{V}_{r}$is

$\hat{V}_{r}=\frac{1}{\sum_{i=1}^{N} w_{ri}^{*}}$ (16).

Here the 95% confidence interval of $\hat{V}_{r}$is obtained based on 1000 Bootstrap samplings.

1. **Clustering of single cell subpopulations**

Suppose that there are *K* methylation states in a given region. As the composition of methylation state is unknown, a mixture model is employed to decompose the mixture methylation states. To this end, we focus on some candidate regions with methylation variation across cells. For a given region$r$, we assume that the proportion of the $k\mathrm{th}$subgroup over the cell population is$\lambda_{rk}$, where$\sum_{k=1}^{K} \lambda_{rk}=1$. As mentioned above, we assume that the number of methylated counts for each CpG site in a given region follows Binomial distribution and in the region the consistent methylation probability follows Beta distribution. Then, we obtain the posterior distribution of methylation probability in the region $r$from cell$i$ $\theta_{ri}$:

$\Pr\left( \theta_{ri} | M_{ri}^{'},N_{ri}^{'} \right)=Beta\left( \sum_{j=1}^{c_{ri}} m_{jri}+\hat{\alpha}_{ri},\sum_{j=1}^{c_{ri}} n_{jri}-\sum_{j=1}^{c_{ri}} m_{jri}+\hat{\beta}_{ri} \right)$,

in which ${{M_{ri}^{'}=(m}_{1ri},m_{2ri},\ldots,m_{c_{ri}ri})}^{T}\mathrm{and}{{N_{ri}^{'}=(n}_{1ri},n_{2ri},\ldots,n_{c_{ri}ri})}^{T}$. Since cells are grouped in the region, the methylation probability of the cells from a subgroup keep consistent. Let $\theta_{r}^{(k)}$denote the methylation probability of group *k*. Then, the probability for the observed methylation in cell *i* is:

$\Pr\left( i \right)=\sum_{k=1}^{K} \Pr\left( k \right)*Pr(i|k)=\sum_{k=1}^{K} \lambda_{rk}\Pr\left( i | \theta_{r}^{(k)} \right)$.

Also, according to the posterior distribution of methylation probability, the conditional probability observed cell $i$ from subgroup $k$ is obtained:

$\Pr\left( i | \theta_{r}^{(k)} \right)=\frac{\Gamma(\sum_{j=1}^{c_{ri}} m_{jri}+\hat{\alpha}_{ri})\Gamma(\sum_{j=1}^{c_{ri}} n_{jri}-\sum_{j=1}^{c_{ri}} m_{jri}+\hat{\beta}_{ri})}{\Gamma(\sum_{j=1}^{c_{ri}} n_{jri}+\hat{\alpha}_{ri}+\hat{\beta}_{ri})}{{(\theta}_{r}^{(k)})}^{\sum_{j=1}^{c_{ri}} m_{jri}+\hat{\alpha}_{ri}-1}*{(1-\theta_{r}^{(k)})}^{\sum_{j=1}^{c_{ri}} n_{jri}-\sum_{j=1}^{c_{ri}} m_{jri}+\hat{\beta}_{ri}-1}$,

where$\Gamma(.)$ is Gamma function.

Therefore, the joint likelihood function:

$L\left( \Theta\right)=\prod_{i=1}^{N} \Pr\left( i \right)$ (17),

where$\Theta=\left( {\lambda_{r1},\lambda_{r2},\ldots,\lambda_{rK}; \theta}_{r}^{\left( 1 \right)}, \theta_{r}^{\left( 2 \right)},\ldots,\theta_{r}^{\left( K \right)} \right)^{T}$. The parameters$\Theta$ may be estimated by maximizing the log likelihood function:

$\hat{\Theta}=\arg\max_{\Theta} logL\left( \Theta\right)=\arg\max_{\Theta} \mathcal{l}\left( \Theta\right)=arg\max_{\Theta} \sum_{i=1}^{N} \log\Pr\left( i \right)$ (18),

The optimized problem may be resolved by Expectation-Maximization (EM) algorithm by a latent random variable $Y_{i}$which denotes the membership of cell *i*: $Y_{i}=k, if cell i is from subgroup k$. Let the probability of $Y_{i}=k$ denote: $Pr(Y_{i}=k)$.

Hence, a rewriting log likelihood is

$\mathcal{l}\left( \Theta\right)=\sum_{i=1}^{N} \log\sum_{k=1}^{K} Pr(Y_{i}=k)\frac{\lambda_{rk}\Pr\left( i | \theta_{r}^{(k)} \right)}{Pr(Y_{i}=k)}\geq\sum_{i=1}^{N} \sum_{k=1}^{K} Pr(Y_{i}=k)\log\frac{\lambda_{rk}\Pr\left( i | \theta_{r}^{(k)} \right)}{Pr(Y_{i}=k)}\triangleq\hbar\left( \Theta\right)$, (19)

In (19), the equality holds if and only if

$\frac{\lambda_{rk}\Pr\left( i | \theta_{r}^{(k)} \right)}{Pr(Y_{i}=k)}=Constant$, (20)

In which we have actually$\sum_{k=1}^{K} \lambda_{rk}=1$and then combined with (20), we obtain a posterior estimation of $Pr(Y_{i}=k)$given the observed cell $i$ and parameters$\Theta$:

$Pr(Y_{i}=k|i, \Theta)=\frac{\lambda_{rk}\Pr\left( i | \theta_{r}^{(k)} \right)}{\sum_{k=1}^{K} \lambda_{rk}\Pr\left( i | \theta_{r}^{(k)} \right)}$. (21)

Then the optimization problem (18) may be estimated by maximizing$\hbar\left( \Theta\right)$, that is,

$\arg\max_{\Theta} \hbar\left( \Theta\right)$ (22). In order to maximize (22), we have

$\left\{ \begin{aligned} \frac{\partial\hbar\left( \Theta\right)}{\partial\lambda_{rk}}=0 \\ \frac{\partial\hbar\left( \Theta\right)}{\partial\theta_{r}^{\left( k \right)}}=0 \end{aligned} \right.$ (23). Consequently, we obtain

$\left\{ \begin{aligned} \lambda_{rk}=\frac{\sum_{i=1}^{N} Pr(Y_{i}=k|i, \Theta)}{N} \\ \theta_{r}^{\left( k \right)}=\frac{\sum_{i=1}^{N} Pr(Y_{i}=k|i, \Theta)(\sum_{j=1}^{c_{ri}} m_{jri}+\hat{\alpha}_{ri}-1)}{\sum_{i=1}^{N} Pr(Y_{i}=k|i, \Theta)(\sum_{j=1}^{c_{ri}} n_{jri}+\hat{\alpha}_{ri}+\hat{\beta}_{ri}-2)} \end{aligned} \right.$, (24) here$k=1, 2,\ldots K$.

In summary, we iteratively estimated all parameters based on EM algorithm. Also, when the function value of $\hbar\left( \Theta\right)$meet the threshold we expected, the iterative process will be stopped,

E-step:

$Pr(Y_{i}=k|i, \Theta)=\frac{\lambda_{rk}\Pr\left( i | \theta_{r}^{(k)} \right)}{\sum_{k=1}^{K} \lambda_{rk}\Pr\left( i | \theta_{r}^{(k)} \right)}$,

M-step:

$\left\{ \begin{aligned} \lambda_{rk}=\frac{\sum_{i=1}^{N} Pr(Y_{i}=k|i, \Theta)}{N} \\ \theta_{r}^{\left( k \right)}=\frac{\sum_{i=1}^{N} Pr(Y_{i}=k|i, \Theta)(\sum_{j=1}^{c_{ri}} m_{jri}+\hat{\alpha}_{ri}-1)}{\sum_{i=1}^{N} Pr(Y_{i}=k|i, \Theta)(\sum_{j=1}^{c_{ri}} n_{jri}+\hat{\alpha}_{ri}+\hat{\beta}_{ri}-2)} \end{aligned} \right.$, here$k=1, 2,\ldots K$.

1. Ziller MJ, Gu H, Muller F, Donaghey J, Tsai LT, Kohlbacher O, et al. Charting a dynamic DNA methylation landscape of the human genome. Nature. 2013;500(7463):477-81.

2. Martuzzi M, Elliott P. Empirical Bayes estimation of small area prevalence of non-rare conditions. Stat Med. 1996;15(17-18):1867-73.
