# Supplementary material for: Integrative single-cell omics analyses reveal epigenetic heterogeneity in mouse embryonic stem cells
Source: PLoS Comput Biol. 2018 Mar 21;14(3):e1006034. doi: 10.1371/journal.pcbi.1006034 (PMC5862410; doi:10.1371/journal.pcbi.1006034)
Supplement: S1 Appendix — (ZIP) [file pcbi.1006034.s011.zip › S1_Appendix/beta_mixture_model/README.docx]

**Beta mixture model**

# Introduction

We developed a beta mixture model to identify putative CSM loci from the candidate CSM regions. Suppose that there are two methylation states which include hyper-methylated state and hypo-methylated state in a given candidate region. Here we assumed that the methylation probabilities of hyper-methylated state and hypo-methylated state are estimated by two distinct the Beta distributions, leading to the distinct θ for two states, with θ^(1)^ and θ^(2)^ indicated methylation probabilities of hyper-methylated and hypo-methylated states, respectively. For each candidate region, the two probabilities were estimated by EM algorithms.

# How to Use

The beta mixture model includes two R scripts, run_beta_mixture_model.R reading in input file and outputting results, and beta_mixture_model.r performing principal statistical analysis, called by run_beta_mixture_model.R. For a better use, simulation.r and the simulated datasets (simul.dat1.txt and simul.dat2.txt) are also provided to perform simulation for evaluating the beta mixture model.

Prepare the input file as the required format, with mandatory values: CHR_ID, START, END, CELL_POSI_INFO in first three and the last columns, respectively.

**INPUT:** the information of methylation statistics of candidate CSM regions

**OUTPUT1:** the result of beta mixture model of candidate CSM regions

**OUTPUT2:** the result of beta mixture model of putative CSM loci

**USAGE:**

**Rscript run_beta_mixture_model.R**

**INPUT FORMAT**

The candidate region file is tab separated and contains following columns:

| CHR_ID* | Chromosome name. |
| --- | --- |
| START* | The start position of candidate region. |
| END* | The end position of candidate region. |
| LEN | The length of candidate region. |
| CELL_COUNT | The counts of cells covering the region. |
| CELL_LIST | The list of cells covering the region, separated by “;”. |
| CELL_INFO | The number of methylated calls, total calls and number of CpG sites of the region in each cell, with the form of “Cell1: #mC_#C_#CpG; Cell2: #mC_#C_#CpG;…”. |
| CELL_POSI_INFO* | The number of methylated calls and total calls of each CpG sites in the region in each cell, with the form of “Cell1: CpGposition1_#mC_#C: CpGposition2_#mC_#C; Cell2: CpGposition1_#mC_#C: CpGposition2_#mC_#C;…”. |

*: Mandatory values

Here’s an example of one candidate region in input file:

| CHR_ID* | chr1 |
| --- | --- |
| START* | 74385316 |
| END* | 74385688 |
| LEN | 373 |
| CELL_COUNT | 5 |
| CELL_LIST | Ser1;Ser11;Ser12;Ser13;Ser20 |
| CELL_INFO | Ser1:14_16_9;Ser11:3_32_8;Ser12:8_10_7;Ser13:8_11_8;Ser20:14_14_7 |
| CELL_POSI_INFO* | **Ser1**:74385410_2_2:74385416_2_2:74385473_1_1:74385488_2_3:74385500_2_2:74385508_2_2:74385510_2_2:74385588_1_1:74385618_0_1;**Ser11**:74385356_0_1:74385410_0_1:74385416_3_3:74385473_0_6:74385488_0_6:74385500_0_7:74385508_0_4:74385510_0_4;**Ser12**:74385410_1_2:74385416_1_2:74385473_2_2:74385488_1_1:74385500_1_1:74385508_1_1:74385510_1_1;**Ser13**:74385356_1_2:74385410_1_2:74385416_2_2:74385473_0_1:74385488_1_1:74385500_1_1:74385508_1_1:74385510_1_1;**Ser20**:74385473_2_2:74385488_2_2:74385500_2_2:74385508_2_2:74385510_2_2:74385588_2_2:74385618_2_2 |

*: Mandatory values

**OUTPUT ARGUMENTS:**

| CHR | Chromosome name. |
| --- | --- |
| START | The start position of candidate region. |
| END | The end position of candidate region. |
| SUBSET1 | Cell(s) grouped into the hyper-methylated cell subset. |
| SUBSET2 | Cell(s) grouped into the hypo-methylated cell subset. |
| MIN.DELTA | The minimum observed methylation difference between cells |
| AVG.M1 | The average methylation level of the predicted hyper-methylated cell subset |
| AVG.M2 | The average methylation level of the predicted hypo-methylated cell subset |
| CELL.NUM | The number of cells covering the candidate region. |
| AVE.METHYLATION | The average methylation level of all cells. |
| LAMMDA | The proportion of the cells with hyper-methylated state in the candidate region. |
| THETA1 | The methylation probability of the hyper-methylated cell subset. |
| THETA2 | The methylation probability of the hypo-methylated cell subset. |
| BIC.PAIR | BIC value under 2 clusters. |
| BIC.SINGLE | BIC value under 1 cluster. |
| CHISQ.VALUE | The value of chi-square. |
| LRT.PVAL | The p-values of likelihood ratio test. |
| VARIANCE | The variance of the methylation level across cells. |
| 2.50% | The variance at the quantile 2.5%. |
| 97.50% | The variance at the quantile 97.5%. |
| LRT.PVAL.ADJUSTED | Adjusted p-values of likelihood ratio test. |

Here’s an example of one region in output file:

| CHR | chr1 |
| --- | --- |
| START | 5018469 |
| END | 5019289 |
| SUBSET1 | "Ser1,Ser13,Ser17,Ser19" |
| SUBSET2 | "Ser12,Ser14,Ser16,Ser2,Ser4,Ser5,Ser9" |
| MIN.DELTA | 0.112429379 |
| AVG.M1 | 0.516720779 |
| AVG.M2 | 0.124286928 |
| CELL.NUM | 11 |
| AVE.METHYLATION | 0.244740967 |
| LAMMDA | 0.363449921 |
| THETA1 | 0.508925072 |
| THETA2 | 0.114738526 |
| BIC.PAIR | 8.936579428 |
| BIC.SINGLE | 59.43551368 |
| CHISQ.VALUE | 55.2947248 |
| LRT.PVAL | 9.84E-13 |
| VARIANCE | 0.000443192 |
| 2.50% | 0.000235843 |
| 97.50% | 0.00070435 |
| LRT.PVAL.ADJUSTED | 2.86E-12 |

# Example analysis

In R console,

## Read in input file

source**(**"beta_mixture_model.r"**)**

# read the input file

# for you own analysis, you will just need to provide the input file with mandatory columns

# mandatory values: CHR_ID, START, END, CELL_POSI_INFO in first three and the last columns, respectively

x **<-** read.table**(**"test.candidate.txt"**)**

# create mat.out for storing the output

mat.out **<-** c**()**

# read the information of one region each time (each row stores one candidate region)

#for(i in 1:nrow(x))

# here we take i=1 (1st region in the 1st row of input file) as an example

i **=** 1

**{**

# read the last column "CELL_POSI_INFO" and store each cell into one element of the vector

cells **<-** strsplit**(**as.character**(**x**[**i,ncol**(**x**)])**, ";"**)[[**1**]]**

# see what the data looks like for region i in cells

# here we take i=1 (1^st^ row) as an example

cells

**[**1**]** "Ser11:3839319_0_5:3839338_0_4:3839356_0_1:3839376_0_2:3839389_0_2:3839442_0_2:3839481_0_3:3839488_0_2:3839520_0_2:3839564_0_4:3839610_0_4"

**[**2**]** "Ser18:3839442_0_1:3839481_3_3:3839488_0_3:3839520_0_3"

**[**3**]** "Ser19:3839087_1_1:3839120_2_2:3839122_2_2:3839134_2_2:3839319_1_1:3839338_2_2:3839356_2_3:3839376_4_4:3839389_3_4:3839442_3_3:3839481_2_2:3839488_2_2:3839520_1_1:3839564_1_1:3839610_0_2:3839659_0_1:3839674_0_1:3839677_1_1:3839693_0_1:3839738_2_2:3839834_0_1"

**[**4**]** "Ser4:3839087_1_1:3839120_2_2:3839122_2_2:3839134_2_2:3839319_1_1:3839338_1_1:3839356_0_1:3839376_1_1:3839389_1_1:3839834_1_1"

**[**5**]** "Ser6:3839120_1_1:3839122_1_1:3839134_1_1:3839319_3_3:3839338_2_2:3839356_0_2:3839376_2_2:3839389_2_2:3839442_0_2:3839481_1_1:3839488_1_1:3839834_0_1"

## Prepare input list for beta_mixture_model function

mat.list **<-** list**(NULL)**; cell.names **<-** c**()**

# read the information of each cell

**for(**j **in** 1**:**length**(**cells**))** **{**

# read the methylation calls of each CpG site in cell j

meth.count **<-** strsplit**(**strsplit**(**cells**[**j**]**, ":"**)[[**1**]]**, "_"**)**

meth.count **<-** unlist**(**meth.count**)**

# the cell.names will store the cell names (5 in this case)

cell.names **<-** c**(**cell.names, meth.count**[**1**])**

# meth.count stores the methylated calls and total calls for each CpG site in region i of cell j

meth.count **<-** meth.count**[**2**:**length**(**meth.count**)]**

meth.count **<-** matrix**(**as.numeric**(**meth.count**)**, length**(**meth.count**)/**3, 3, byrow**=**T**)[**,2**:**3**]**

# the mat.list will store the meth.count for all cells

**if(**is.null**(**mat.list**[[**1**]]))** mat.list **<-** list**(**meth.count**)** **else** mat.list **<-** c**(**mat.list, list**(**meth.count**))**

**}**

# see what the data looks like for cell j of region i in meth.count, here we take the last cell (j=5 in this case, that is 5^th^ cell, Ser6) as an example

# rows represent all CpG sites in region i of cell j, columns represent the methylated calls and total calls

meth.count

**[**,1**]** **[**,2**]**

**[**1,**]** 1 1

**[**2,**]** 1 1

**[**3,**]** 1 1

**[**4,**]** 3 3

**[**5,**]** 2 2

**[**6,**]** 0 2

**[**7,**]** 2 2

**[**8,**]** 2 2

**[**9,**]** 0 2

**[**10,**]** 1 1

**[**11,**]** 1 1

**[**12,**]** 0 1

names**(**mat.list**)** **<-** cell.names

# check the cells of region i in cell.names

# example of i=1, 5 cells in this case

cell.names

**[**1**]** "Ser11" "Ser18" "Ser19" "Ser4" "Ser6"

# see what the data looks like for the first element of region i in mat.list (Ser11 in this case)

mat.list**[**1**]**

**$**Ser11

**[**,1**]** **[**,2**]**

**[**1,**]** 0 5

**[**2,**]** 0 4

**[**3,**]** 0 1

**[**4,**]** 0 2

**[**5,**]** 0 2

**[**6,**]** 0 2

**[**7,**]** 0 3

**[**8,**]** 0 2

**[**9,**]** 0 2

**[**10,**]** 0 4

**[**11,**]** 0 4

## Call beta_mixture_model function

# analyze the region i by performing beta_mixture_model function

output **<-** unlist**(**beta_mixture_model**(**mat.list**))**

# see what the data looks like in output after running beta mixture model

output

subset1 subset2 min.delta

"Ser19,Ser4,Ser6" "Ser11,Ser18" "0.436842105263158"

avg.m1 avg.m2 cell.num

"0.818263607737292" "0.15" "5"

avg.methylation lammda theta1

"0.459131803868646" "0.606748990526955" "0.809058210645288"

theta2 BIC.pair BIC.single

"0.0712883257042949" "3.73962955540628" "57.5854339560656"

chisq.value LRT.pval variance

"57.0646802255275" "4.06033749155989e-13" "0.00149159733739751"

# calculate the confidence interval of methylation variance and combined to output

ci **<-** quantile**(**variance_boot_ci**(**mat.list**)**, c**(**0.025, 0.975**))**

mat.out **<-** rbind**(**mat.out, c**(**output, ci**))**

**}**

# formatting and outputting

label **<-** colnames**(**mat.out**)**

rownames**(**mat.out**)** **<-** rownames**(**x**)**

# calculate adjusted p-value and combined to output, and add the first three columns "CHR_ID", "START", and "END" into output

mat.out **<-** cbind**(**x**[**,1**:**3**]**, mat.out, p.adjust**(**as.numeric**(**paste**(**mat.out**[**,14**]))**, method**=**"BH"**))**

colnames**(**mat.out**)** **<-** c**(**"chr", "start", "end", label, "LRT.pval.adjusted"**)**

# for your own analysis, replace the output file name

write.table**(**mat.out, file**=**"test.candidate.BetaMixtureModelResult.txt", quote**=**F, sep**=**"\t", row.names**=**F, col.names**=**T**)**

## Produce putative CSM loci

# get csm with the following cutoffs:

# 1, valid cluster1 and cluster2: avg.m1 != -1; avg.m2 != -1

# 2, observed minimum methylation difference of two methylation states: min.delta >0.1

# 3, threshold of methylation difference of two methylation states: theta1–theta2 >=0.3

# 4, required number of cells: cell.num >=8

# 5, adjusted p-value: LRT.pval.adjusted <0.05

mat.out **<-** read.table**(**"test.candidate.BetaMixtureModelResult.txt",h**=**T,stringsAsFactors**=FALSE)**

x.out **=** mat.out**[**mat.out**$**min.delta **>** 0.1 **&** mat.out**$**avg.m1 **!=** **-**1 **&** mat.out**$**avg.m2 **!=** **-**1 **&** mat.out**$**cell.num **>=** 8 **&** mat.out**$**LRT.pval.adjusted **<** 0.05 **&** mat.out**$**theta1 **-** mat.out**$**theta2 **>=** 0.3,**]**

# for your own analysis, replace the output file name

write.table**(**x.out, file**=**"test.csm.txt", quote**=**F, sep**=**"\t", row.names**=**F, col.names**=**T**)**
